# Control of Heterologous Simian Immunodeficiency Virus SIV_smE660_ Infection by DNA and Protein Coimmunization Regimens Combined with Different Toll-Like-Receptor-4-Based Adjuvants in Macaques

**DOI:** 10.1128/JVI.00281-18

**Published:** 2018-07-17

**Authors:** Shakti Singh, Eric G. Ramírez-Salazar, Rami Doueiri, Antonio Valentin, Margherita Rosati, Xintao Hu, Brandon F. Keele, Xiaoying Shen, Georgia D. Tomaras, Guido Ferrari, Celia LaBranche, David C. Montefiori, Jishnu Das, Galit Alter, Hung V. Trinh, Christopher Hamlin, Mangala Rao, Frances Dayton, Jenifer Bear, Bhabadeb Chowdhury, Candido Alicea, Jeffrey D. Lifson, Kate E. Broderick, Niranjan Y. Sardesai, Sandra J. Sivananthan, Christopher B. Fox, Steven G. Reed, David J. Venzon, Vanessa M. Hirsch, George N. Pavlakis, Barbara K. Felber

**Affiliations:** a Human Retrovirus Pathogenesis Section, Vaccine Branch, Center for Cancer Research, National Cancer Institute at Frederick, Frederick, Maryland, USA; b Human Retrovirus Section, Vaccine Branch, Center for Cancer Research, National Cancer Institute at Frederick, Frederick, Maryland, USA; c AIDS and Cancer Virus Program, Leidos Biomedical Research, Inc., Frederick National Laboratory for Cancer Research, Frederick, Maryland, USA; d Duke Human Vaccine Institute, Department of Medicine, Duke University Medical Center, Durham, North Carolina, USA; e Duke Human Vaccine Institute, Department of Immunology, Duke University Medical Center, Durham, North Carolina, USA; f Duke Human Vaccine Institute, Department of Molecular Genetics, Duke University Medical Center, Durham, North Carolina, USA; g Duke Human Vaccine Institute, Department of Surgery, Duke University Medical Center, Durham, North Carolina, USA; h Ragon Institute of MGH, MIT, and Harvard University, Cambridge, Massachusetts, USA; i U.S. Military HIV Research Program, Walter Reed Army Institute of Research, Silver Spring, Maryland, USA; j Henry M. Jackson Foundation for the Advancement of Military Medicine, Bethesda, Maryland, USA; k Inovio Pharmaceuticals, Inc., Plymouth Meeting, Pennsylvania, USA; l Infectious Disease Research Institute, Seattle, Washington, USA; m Biostatistics and Data Management Section, Center for Cancer Research, National Cancer Institute, National Institutes of Health, Rockville, Maryland, USA; n Laboratory of Molecular Microbiology, National Institute of Allergy and Infectious Diseases, National Institutes of Health, Bethesda, Maryland, USA; Ulm University Medical Center

**Keywords:** DNA, protein, TLR4, TLR7, QS21, adjuvant, rhesus macaque, vaccination, vaccine, immunization, SIV_mac251_, SIV_smE660_, HIV, SIV_smE660_ T/F, A/K variant, TRIM-5α, humoral responses, binding antibody, neutralizing antibody, linear peptide, cyclic V2, scaffolded gp70-V1V2, ADCC, ADCD, ADNP, Ab glycosylation structures, T cell responses, mucosal responses, repeated low-dose rectal challenge, reduced risk of infection, viremia control, correlate of viremia control, V2 responses, acquisition delay, systems serology

## Abstract

We developed a method of simultaneous vaccination with DNA and protein resulting in robust and durable cellular and humoral immune responses with efficient dissemination to mucosal sites and protection against simian immunodeficiency virus (SIV) infection. To further optimize the DNA-protein coimmunization regimen, we tested a SIV_mac251_-based vaccine formulated with either of two Toll-like receptor 4 (TLR4) ligand-based liposomal adjuvant formulations (TLR4 plus TLR7 [TLR4+7] or TLR4 plus QS21 [TLR4+QS21]) in macaques. Although both vaccines induced humoral responses of similar magnitudes, they differed in their functional quality, including broader neutralizing activity and effector functions in the TLR4+7 group. Upon repeated heterologous SIV_smE660_ challenge, a trend of delayed viral acquisition was found in vaccinees compared to controls, which reached statistical significance in animals with the TRIM-5α-resistant (TRIM-5α R) allele. Vaccinees were preferentially infected by an SIV_smE660_ transmitted/founder virus carrying neutralization-resistant A/K mutations at residues 45 and 47 in Env, demonstrating a strong vaccine-induced sieve effect. In addition, the delay in virus acquisition directly correlated with SIV_smE660_-specific neutralizing antibodies. The presence of mucosal V1V2 IgG binding antibodies correlated with a significantly decreased risk of virus acquisition in both TRIM-5α R and TRIM-5α-moderate/sensitive (TRIM-5α M/S) animals, although this vaccine effect was more prominent in animals with the TRIM-5α R allele. These data support the combined contribution of immune responses and genetic background to vaccine efficacy. Humoral responses targeting V2 and SIV-specific T cell responses correlated with viremia control. In conclusion, the combination of DNA and gp120 Env protein vaccine regimens using two different adjuvants induced durable and potent cellular and humoral responses contributing to a lower risk of infection by heterologous SIV challenge.

**IMPORTANCE** An effective AIDS vaccine continues to be of paramount importance for the control of the pandemic, and it has been proven to be an elusive target. Vaccine efficacy trials and macaque challenge studies indicate that protection may be the result of combinations of many parameters. We show that a combination of DNA and protein vaccinations applied at the same time provides rapid and robust cellular and humoral immune responses and evidence for a reduced risk of infection. Vaccine-induced neutralizing antibodies and Env V2-specific antibodies at mucosal sites contribute to the delay of SIV_smE660_ acquisition, and genetic makeup (TRIM-5α) affects the effectiveness of the vaccine. These data are important for the design of better vaccines and may also affect other vaccine platforms.

## Abstract (precis)

An effective AIDS vaccine continues to be of paramount importance for the control of the pandemic, and it has been proven to be an elusive target. Vaccine efficacy trials and macaque challenge studies indicate that protection may be the result of combinations of many parameters. We show that a combination of DNA and protein vaccinations applied at the same time provides rapid and robust cellular and humoral immune responses and evidence for a reduced risk of infection. Vaccine-induced neutralizing antibodies and Env V2-specific antibodies at mucosal sites contribute to the delay of SIV_smE660_ acquisition, and genetic makeup (TRIM-5α) affects the effectiveness of the vaccine. These data are important for the design of better vaccines and may also affect other vaccine platforms.

## INTRODUCTION

The development of a vaccine against human immunodeficiency virus (HIV) remains an important research aim since only the RV144 trial showed marginal protection against infection. The prime-boost vaccine used in the RV144 trial is comprised of recombinant ALVAC-expressing genes coding for Gag/protease and membrane-bound gp120 Env and AIDSVAX gp120 protein subtypes CRF01_AE and clade B (MN) adjuvanted in alum (1). Importantly, analysis of the RV144 data suggested that the development of nonneutralizing antibody (Ab) responses, including responses to variable region 2 (V2) of HIV Env, and antibody-dependent cellular cytotoxicity (ADCC) were associated with a lower risk of infection (1–4). This vaccine failed to induce durable responses and had marginal efficacy.

We have developed candidate DNA vaccines against HIV/simian immunodeficiency virus (SIV) that induce immune responses able to efficiently reduce viremia in different SIV challenge models for prevention (5–8) and therapy (9, 10). DNA as a vaccine platform has several advantages, including simplicity, scalability, and the possibility for repeated boosts due to the lack of immunity against the vector. The combination of intramuscular (i.m.) DNA delivery followed by *in vivo* electroporation (EP) has been shown to be a more effective vaccine delivery method, inducing higher immune responses in macaques and in humans (reviewed in references 11 and 12). To improve antibody development, we had previously included protein as a boost in classical prime-boost vaccination or employed a coimmunization regimen of DNA and protein, termed DNA-protein coimmunization, delivered simultaneously at the same anatomical site (7, 13–16). The coimmunization vaccine regimen resulted in higher and more durable antibody responses with improved mucosal dissemination and robust systemic and mucosal T cell responses (7, 13–17). In a previous DNA-protein coimmunization study, we used aldrithiol-2 (AT-2)-inactivated SIV_mac239_ viral particles as a protein component and reported a significant delay in the acquisition of the heterologous SIV_smE660_ (7). The DNA-protein coimmunization vaccine regimen has also been used by other groups and resulted in improved antibody responses in rabbits and macaques (18–21).

The design of an efficient vaccine regimen requires optimization of the immunogens and additional components, including adjuvants. The inclusion of adjuvants like QS21 (22–25), agonists of specific Toll-like receptors (TLRs) (13, 14, 26–42), or combinations thereof (43–46) has generated strong interest due to their ability to enhance immune responses. Recent studies have indicated that such adjuvants can act as strong enhancers of immune responses, resulting in various degrees of protection from SIV infection (31, 42, 46). We have previously shown that Env protein adjuvanted with the TLR4 agonist formulated in a stable oil-in-water emulsion (GLA-SE) and coimmunized with DNA provided higher titers of durable antibody responses against HIV and SIV Env (13, 14). In the present study, we expanded the use of TLR4-based adjuvants in combination with SIV DNA-protein coimmunization regimens by comparing two liposomal adjuvant formulations, TLR4 plus TLR7 (TLR4+7) and TLR4 plus QS21 (TLR4+QS21). We tested vaccine efficacy upon repeated low-dose rectal heterologous SIV_smE660_ challenge. Tripartite-motif-containing protein 5 alpha (TRIM-5α) (47–49) is a potent innate restriction factor which affects SIV_smE660_ infection (50–53), exerting its function via interaction with the incoming virus particles, in particular with the viral capsid protein, a proteolytic Gag-processing product, and it was shown that mutations in two sites (P37S and R98S) in the capsid protein could alleviate this antiviral effect (52). The TRIM-5α-resistant (TRIM-5α R) allele as an innate protective immune mechanism can assist vaccine-induced immune responses in controlling infection (31, 54–56). Balancing the groups, we also addressed the contribution of the TRIM-5α genotype in our study.

## RESULTS

### DNA-protein vaccination regimens using different protein adjuvants in rhesus macaques.

**FIG 1:**
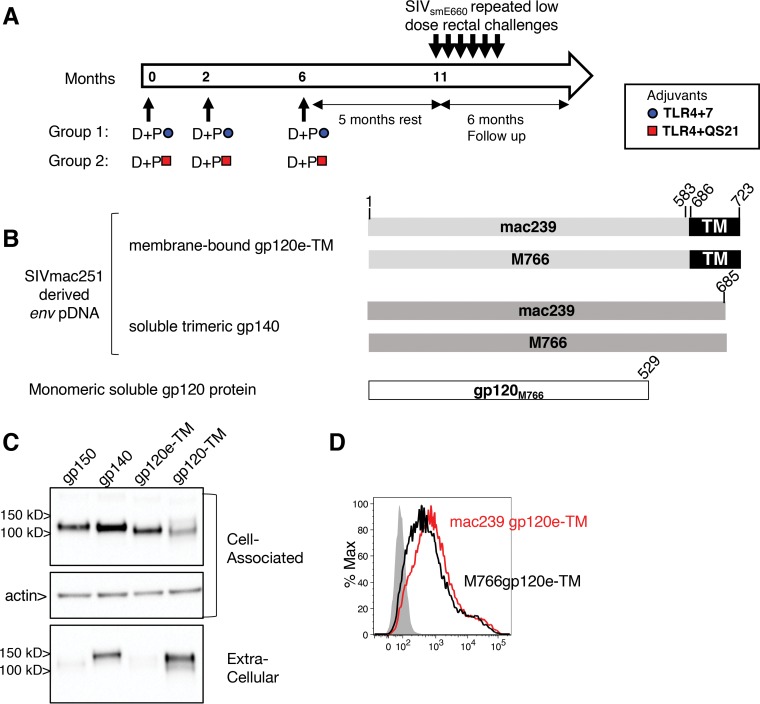
Vaccination of macaques with SIV_mac251_ DNA-protein coimmunization vaccine regimens. (A) Indian rhesus macaques were vaccinated three times (0, 2, and 6 months) with SIV_mac251_-derived *env* plasmids (SIV_mac239_ and T/F M766) coadministered with monomeric M766 gp120 protein adjuvanted with TLR4+7 (*n* = 12) or TLR4+QS21 (*n* = 12). The DNA mixtures also contained SIV_mac239_ *gag* DNA and rmIL-12 DNA. Five months after the 3rd vaccination, the animals were subjected to weekly intrarectal exposures using a titrated dose of the heterologous SIV_smE660_ virus, and the infected animals were monitored for 6 months. (B) Schematic representation of the DNA-protein vaccine comprising RNA/DNA-optimized expression vectors producing SIV_mac251_-derived membrane-bound gp120e-TM and the soluble trimeric gp140 Env proteins. The vaccine contained monomeric M766 gp120 Env. Amino acid positions follow SIV_mac239_ numbering. (C) HEK293T cells were transfected with SIV M766 *env* plasmid DNAs (pDNAs) expressing gp150 (lane 1), gp140 (lane 2), gp120e-TM (lane 3), and gp120-TM (lane 4). Proteins from the cell-associated and extracellular (1/200 of each sample) fractions were analyzed by Western immunoblotting and detected using a mouse anti-gp120 Ab. Equal loading of the blot with the cell-associated fractions was controlled by probing the membrane with an antiactin antibody. (D) Histogram overlay showing the membrane localization of mac239 and M766 gp120e-TM proteins on transfected HEK293 cells using a mouse anti-gp120 Ab followed by an APC-conjugated goat anti-mouse Ab. The mock-transfected cells are shown (gray histogram).

Indian rhesus macaques (*n* = 12/vaccine group; 12 controls) were balanced for age (median, 2.9 years) and weight (median, 3.6 kg) and comprised 5 females per vaccine group and 6 females in the control group. The groups were balanced for TRIM-5α alleles: either resistant (referred to as R here) (TFP/TFP; TFP/CYPA) or moderate (TFP/Q; Q/CYPA)/sensitive (Q/Q) (referred to as M/S here), with respect to permissiveness for SIV_smE660_ infection (50–53) (see Table S1 in the supplemental material). Two groups received SIV DNA-protein coimmunization vaccine regimens (Fig. 1A) differing only by the adjuvants used for protein formulations. Both groups received a DNA vaccine comprising a mixture of SIV_mac251_ Env sequences from the transmitted/founder (T/F) SIV_mac251_ M766 and the infectious molecular clone SIV_mac239_, which differ by 5% of amino acids over the complete gp160 sequence. The DNA plasmids expressed two forms of Env (Fig. 1B): (i) soluble trimeric gp140 and (ii) membrane-bound gp120e-TM, a fusion of gp120 to the transmembrane (TM) region. In contrast to the HIV gp120-TM *env* insert in rALVAC used in the RV144 trial (1), gp120e-TM consists of gp120 with an additional 54 C-terminal amino acids spanning the fusion peptide and part of the heptad region. Upon transient transfection, the gp120e-TM protein was found to be mainly cell associated, whereas the Env protein produced from gp120-TM Env was efficiently secreted (Fig. 1C). Flow cytometry analysis confirmed that mac239 and M766 gp120e-TM proteins are exposed at the cell surface (Fig. 1D). The DNA vaccine mixture also contained plasmids expressing SIV Gag and rhesus macaque interleukin-12 (rmIL-12), as a molecular adjuvant, which we and others previously showed to increase vaccine-induced immunity in macaques (16, 57–63). The DNA vaccine was coadministered with HEK293 cell-produced the soluble monomeric SIV M766 (64, 65) gp120 Env protein, adjuvanted with liposomal formulations containing a combination of TLR4 and TLR7 agonists (referred to as TLR4+7 here) or with a combination of a TLR4 agonist and QS21, a saponin derivative (referred to as TLR4+QS21 here). Six animals from the control group received 3 sham DNA and IL-12 DNA vaccinations, which included the respective adjuvants at the last vaccination (*n* = 3 each). Six treatment-naive animals were included in the control group. Vaccinations were performed at 0, 2, and 6 months (Fig. 1A). The DNA-protein coimmunization vaccine was administered via the intramuscular route by *in vivo* EP of the DNA, followed immediately by the administration of the protein vaccine into the same muscle.

The health of the animals was regularly monitored by physical examination, complete blood count (CBC), and serum chemistry. No adverse effects were observed in either adjuvant-treated or control animals over the course of the study. In addition, changes in lymphocyte activation related to the different adjuvants used in the vaccine were monitored in blood CD4^+^ and CD8^+^ T cells. These analyses included determining the expression of granzyme B (GrzB) (cytotoxicity), Ki67 (cell cycling), CXCR3 (effector T cell trafficking and function), α4β7 (gut-homing receptor associated with HIV infection) (66–68), and major histocompatibility complex class II (MHC-II) (T cell activation) and showed no differences among animals in the vaccine or control groups (Fig. S1).

### DNA-protein vaccines induce robust systemic and mucosal humoral immune responses, including V2-specific bAb.

**FIG 2:**
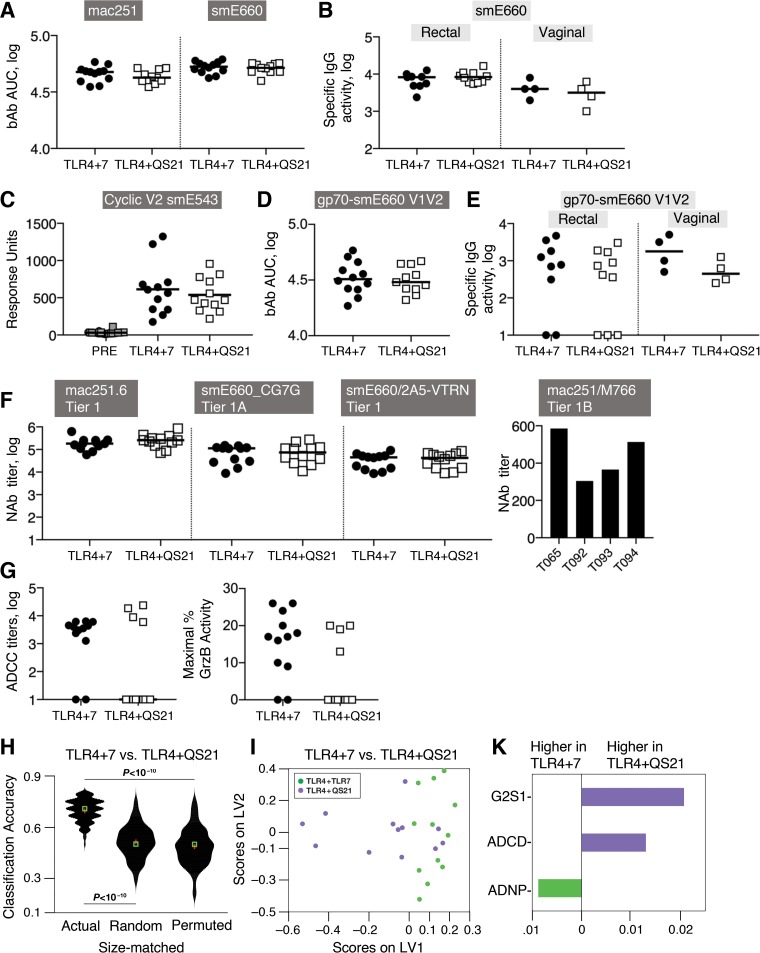
Systemic and mucosal humoral immune responses induced by the DNA-protein coimmunization vaccine. Humoral responses were measured 2 weeks after the 3rd vaccination (bars indicate median values). (A) bAb to SIV_mac251_ and SIV_smE660_ in plasma determined by SIV-BAMA are shown as the area under the curve of the binding magnitude (AUC). (B) gp140_smE660_-specific binding activity measured by SIV-BAMA in rectal and vaginal (samples from 4 of the 5 females could be analyzed) mucosal samples are shown as Env-specific binding antibody (MFI × dilution/total IgG). (C) Responses in plasma to cyclic V2 were measured to SIV_smE543_ V2 peptide (aa 168 to 206) by a SPR assay. (D and E) bAb recognizing gp70-scaffolded SIV_smE660_-specific V1V2 measured in plasma (D) and in mucosal (rectal and vaginal) samples (E) using SIV-BAMA. (F) NAb to SIV_mac251.6_, SIV_smE660_ (BR-CG7G.IR1 and 2A5-VTRN), and SIV_mac251_ M766 in plasma. Titers are calculated as 50% infectious doses (ID_50_) (dilution) in TZM-bl cells with a threshold of 300. (G) Serially diluted serum samples were used to determine the ADCC titers (left) and peak granzyme B activity (right) using SIV_mac251_ gp120-coated CEM-NKR target cells. (H to K) Systems serology shows distinct function and Ab glycoforms. (H) Violin plot illustrating the performance of the actual model and 2 negative-control null models (random features and permuted data) for comparison of the two vaccine groups. The violin plot illustrates the distribution of the classification accuracies of the actual and null models, as measured across 100 independent 5-fold cross-validation replicates. (I) Scores plot of a LASSO/PLS model illustrating separation between animals from the 2 vaccine groups. (K) Variable importance in projection (VIP) plot showing the variables that were identified by the model distinguishing the animals in each vaccine groups. The length of the bar corresponds to the relative importance of the variable, and the color of the bar corresponds to which arm the variable is higher.

Both DNA-protein vaccines induced binding antibody (bAb) recognizing vaccine-matched SIV_mac251_ and heterologous SIV_smE660_ (Fig. 2A), and those IgG antibodies efficiently disseminated into rectal and vaginal mucosa (Fig. 2B) in animals from both vaccine groups. We tested the ability of the vaccine-induced bAb to recognize the V2 region, since V2-specific Ab were associated with a delay in virus acquisition in the RV144 clinical trial (1–3) and in an analogous SIV vaccine study performed in macaques (69, 70). Of note, amino acid alignment of the vaccine-matched SIV_mac251_ Env sequences mac239/M766 and the heterologous SIV_smE660_ sequence shows 62% identity of the V1V2 regions, with 90% identity of the V2 regions (see Fig. S2A in the supplemental material). Plasma samples collected 2 weeks after the 3rd vaccination were analyzed for reactivity against (i) linear peptides covering amino acids (aa) 16 to 717 (amino acid numbering according to SIV_mac239_) of Env (Fig. S2B and S3) and (ii) cyclic V2 (Fig. 2C and Fig. S2C) to identify interactions with constrained peptides mimicking some of the conformations of the V2 region. In addition, the presence of antibodies recognizing scaffolded gp70-V1V2 (Fig. 2D and E and Fig. S2D and S2E) was also analyzed in both plasma and mucosal secretions to detect interactions with V1V2 in a structural context. The plasma bAb in the animals from both vaccine groups recognized primarily V1 (maximum response to peptides 16 and 17) and V2 (maximum response to peptides 26, 27, and 28) and, to a lesser extent, C3 (maximum response to peptides 57 and 58), V4 (maximum response to peptides 67 and 68), and C5 (maximum response to peptides 82 and 83) (Fig. S3). Low responses to V3 were found, in agreement with our previous observation that SIV DNA-protein-vaccinated macaques develop stronger antibody responses to V1 and V2 (14, 71), a finding also reported for other SIV vaccine platforms (54, 71). To allow direct comparison among the animals, the antibody responses targeting the different Env regions were estimated as percentages of the total anti-Env response for each animal (Fig. S2B). This analysis showed significantly higher responses targeting V2 (*P* = 0.0077) and lower responses to C3 (*P* < 0.0001) in the animals from the TLR4+7 group.

To further explore the recognition of the V2 region, plasma samples were tested for their reactivity to cyclic V2 peptides. Both vaccine groups showed robust binding to cyclic V2 from SIV_mac251_ (Fig. S2C) and SIV_smE543_ (Fig. 2C). No difference was found between responses to cyclic peptides spanning aa 151 to 206 full length (F) and aa 168 to 206 short length (S), indicating that the recognized epitope(s) lies within the shorter peptide spanning aa 168 to 206, a region overlapping the sequence covered by linear peptides 26 to 28 (aa 168 to 194) (Fig. S2A). We also found robust recognition of both scaffolded vaccine-matched gp70-SIV_mac251_ V1V2 (Fig. S2D) and heterologous gp70-SIV_smE660_ V1V2 (Fig. 2D). Importantly, analysis of rectal and vaginal secretion samples showed the presence of antibodies binding to V1V2, demonstrating efficient mucosal dissemination of the vaccine-induced humoral responses in both groups of vaccinees (Fig. 2E and Fig. S2E). We also measured mucosal SIV_mac251_-specific IgA and found responses in only two animals (T093 and T094) from the TLR4+7 group. Macaque T094 was also positive for IgA antibodies targeting V1V2. Taken together, these data demonstrate that the humoral responses induced by our vaccine regimen efficiently disseminate to mucosa rather than local antibody production, in accord with our previous report (14).

In summary, the two DNA-protein vaccine formulations induced robust humoral responses recognizing the vaccine-matched and heterologous V2 regions, both in the context of linear peptides and conformational epitopes (cyclic V2 and the gp70 scaffold), and, specifically, recognizing the N-terminal portion of V2, which shares the highest homology between SIV_mac251_ and SIV_smE660_ (Fig. S2A).

### Distinct antibody functions between the vaccine groups.

Vaccinees were evaluated for their ability to mount neutralizing antibody (NAb) to the vaccine-matched SIV_mac251_ and the heterologous SIV_smE660_ (Fig. 2F). Macaques in both vaccine groups developed robust levels of NAb to tier 1 SIV_mac251.6_, tier 1A SIV_smE660_-CG7G, as well as neutralization-sensitive tier 1 SIV_smE660_/2A5-VTRN (Fig. 2F). Of note, 4 of the 12 animals in the TLR4+7 group (animals T065, T092, T093, and T094) showed NAb to tier 1B SIV_mac251_ M766 (Fig. 2F, right), indicating the induction of broader NAb responses using this adjuvant formulation. No neutralizing activity was found against tier 2 SIV_mac251.41_ and neutralization-resistant tier 2 SIV_smE660_ CR54-PK-2A5. Monitoring the persistence of neutralizing activity after the 3rd vaccination showed that NAb, like bAb (see Fig. S4A in the supplemental material), had similar durabilities among the two groups, with an ∼1-log decline over 5 months (Fig. S4B).

We also evaluated whether the antibodies induced by the two vaccine formulations were able to induce cell-mediated cytotoxicity (ADCC) using (i) SIV_mac251_ gp120-coated target cells and (ii) SIV_mac251_- and SIV_smE660_-infected target cells. Figure 2G shows (i) ADCC endpoint titers (defined as the reciprocal of the highest dilution indicating a positive response) (left) and (ii) maximum percent GrzB activity (defined as the maximum percent frequency of GrzB-positive [GrzB^+^] cells observed at any plasma dilution) (right). Both assays measure ADCC by different final readouts, and the similar results obtained by both assays support the conclusion that more animals were positive for ADCC in the TLR4+7 vaccine group (10 of 12) than in the TLR4+QS21 group (4 of 12). Finally, we detected ADCC activity against the SIV_mac251_-infected cells in only one immunized animal (macaque T065 from the TLR4+7 group).

Taken together, these data showed that, despite the similar magnitudes of the humoral responses induced by the two vaccines, macaques in the TLR4+7 group developed more functional responses mediated by antibodies, including ADCC and broader neutralizing activity.

### System serology approaches to interrogate group-specific differences in vaccinated animals.

We used a comprehensive systems approach to survey an array of antibody features and functions (72–75), as an additional way to interrogate differences between the two vaccine groups (Fig. 2H, I, and K). We profiled seven Fc effector functions, three IgG-mediated functions pertaining to the activation of natural killer (NK) cells (CD107a/degranulation, interferon gamma [IFN-γ], and macrophage inflammatory protein 1β [MIP-1β] secretion), ADCD (antibody-dependent complement deposition), ADCC, ADCP (antibody-dependent cellular phagocytosis) (i.e., mediated by monocytes), and ADNP (antibody-dependent neutrophil phagocytosis), and comprehensively characterized the associated IgG glycosylation profile of the M766-specific Ab (see Table S2 in the supplemental material).

We employed a composite multivariate model based on the least absolute shrinkage and selection operator (LASSO) and partial least-squares (PLS) discriminant analysis (PLSDA) (Fig. 2H) to dissect differences between the vaccine groups (Fig. 2I and K). The adjuvants induced functional and glycan differences across the two groups: while higher ADNP was associated with the TLR4+7 group, ADCD and the G2S1 (digalactosylated [G2], sialylated [S1]) glycoforms were associated with the TLR4+QS21 group (Fig. 2K). Thus, the same SIV DNA-protein vaccine, which differs only in the included adjuvant, led to distinct profiles of antibody effector functions and antibody glycoforms.

### Vaccinees with a TRIM-5α-resistant genotype show a delay in SIV_smE660_ acquisition.

Five months after the 3rd vaccination, the animals were challenged by weekly intrarectal low-dose exposures using the same well-characterized stock of the heterologous SIV_smE660_ used in previous studies (7, 76–78). The SIV_smE660_ stock, grown in macaque lymphocytes, comprises an uncloned virus swarm and differs by 20% in the Env amino acid sequence from the SIV_mac251_ Env used in the vaccine, reflecting the cross-clade breadth of HIV strains.

**FIG 3:**
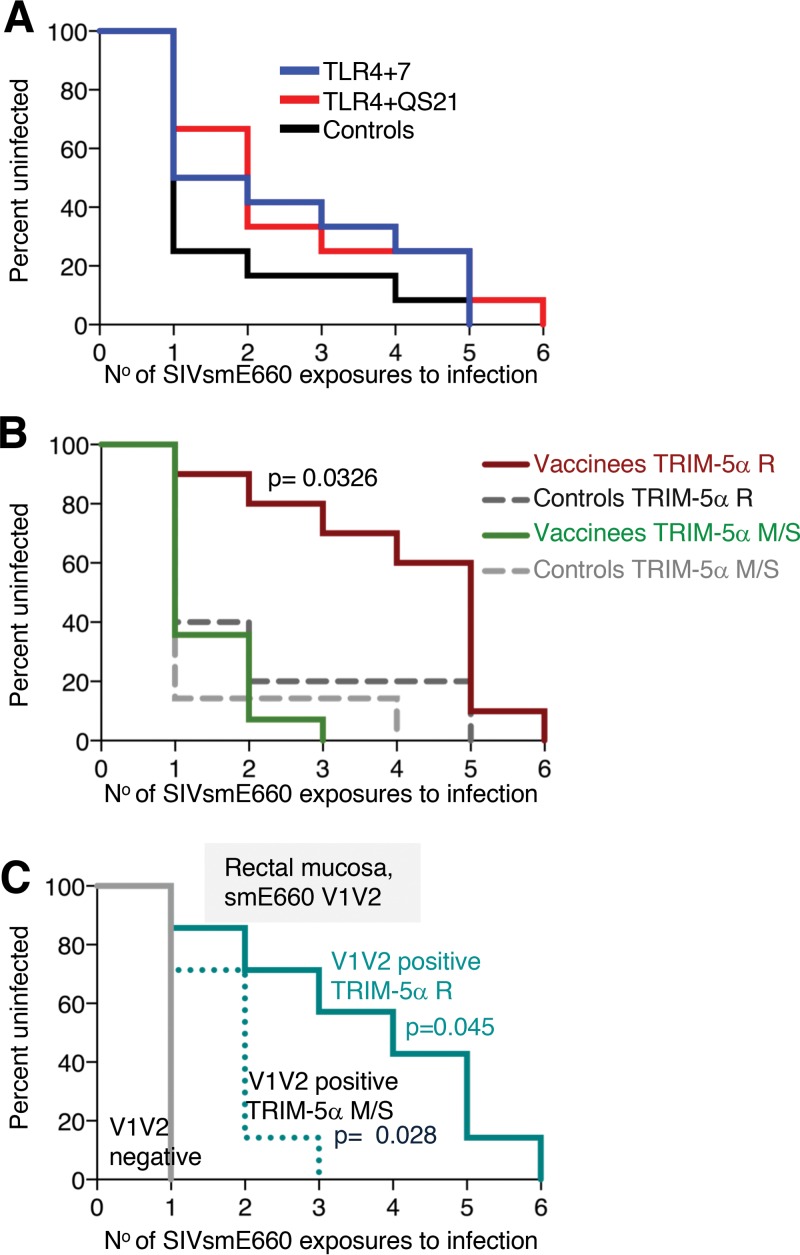
Delay in SIV_smE660_ acquisition in vaccinees. (A and B) Kaplan-Meier curves of the number of SIV_smE660_ challenges for infection of the two vaccine groups (*n* = 12 each) and the control (*n* = 12) (A) and the combined group of animals with the TRIM-5α R genotype (vaccinees, *n* = 10) and controls (*n* = 5) and of animals with the TRIM-5α M/S genotype (vaccinees, *n* = 14) and controls (*n* = 7) (B). *P* values comparing vaccinees and controls with the TRIM-5α R genotype are from a Gehan-Breslow-Wilcoxon test. (C) Vaccinees with positive rectal V1V2 responses and carrying the TRIM-5α R or TRIM-5α M/S allele are compared to V1V2-negative animals. *P* values are from an exact log rank test.

After an initial delay compared to the control group, all vaccinees became infected by the 6th exposure (Fig. 3A). The delay in virus acquisition of the two vaccine groups did not reach statistical significance compared to macaques in the control group. To examine the contribution of host genetics to the vaccine-induced protective responses, we evaluated the role of the TRIM-5α genotype, reported to influence the permissiveness for SIV_smE660_ acquisition and replication (50–54). Comparing only animals with the TRIM-5α R allele (combined vaccine groups [*n* = 10] versus controls [*n* = 5]), we found a significant delay in virus acquisition in the TRIM-5α R vaccinees (Fig. 3B) (*P* = 0.0326 by a Gehan-Breslow-Wilcoxon test). No difference in the acquisition rate was found comparing the animals carrying the TRIM-5α M/S genotype. Comparison of TRIM-5α R animals of the individual vaccine groups with TRIM-5α R controls showed delays in acquisition in both groups, and the TLR4+QS21 group reached significance (*P* = 0.0468 by a Gehan-Breslow-Wilcoxon test) (see Fig. S5 in the supplemental material).

Examining the contribution of humoral responses to the delay of virus acquisition, we found that animals having rectal V1V2-specific responses to SIV_smE660_, but not SIV_mac251_, showed a significant delay in infection (Fig. 3C). Animals with positive rectal V1V2 responses and having the TRIM-5α M/S genotype have a significant delay in virus acquisition compared to the animals with no V1V2-specific responses (*P* = 0.028 by an exact log rank test), indicating a vaccine effect in the TRIM-5α M/S animals. In addition, among animals with positive rectal V1V2 responses, there is a significant delay comparing animals having the TRIM-5α R genotype versus animals having the TRIM-5α M/S genotype (*P* = 0.045 by an exact log rank test). Thus, a combination of vaccine-induced immune responses and genetic background contributed to the delay in SIV_smE660_ infection in both vaccine groups.

### Vaccine-induced sieve effect selects for neutralization-resistant SIV_smE660_ T/F variants.

We next interrogated the number and genetic makeup of distinct T/F variants using single-genome amplification (SGA) and direct sequencing of the T/F *env* genes from each plasma sample collected at the peak of primary viremia (2 to 3 weeks postinfection). The inferred amino acid sequences of individual lineages representing the infecting T/F genome were determined. Phylogenetic analysis of the Env sequences from T/F variants did not show any clustering and showed a distribution of sequences similar to that of the swarm (79) found in a previous infection study (7).

**FIG 4:**
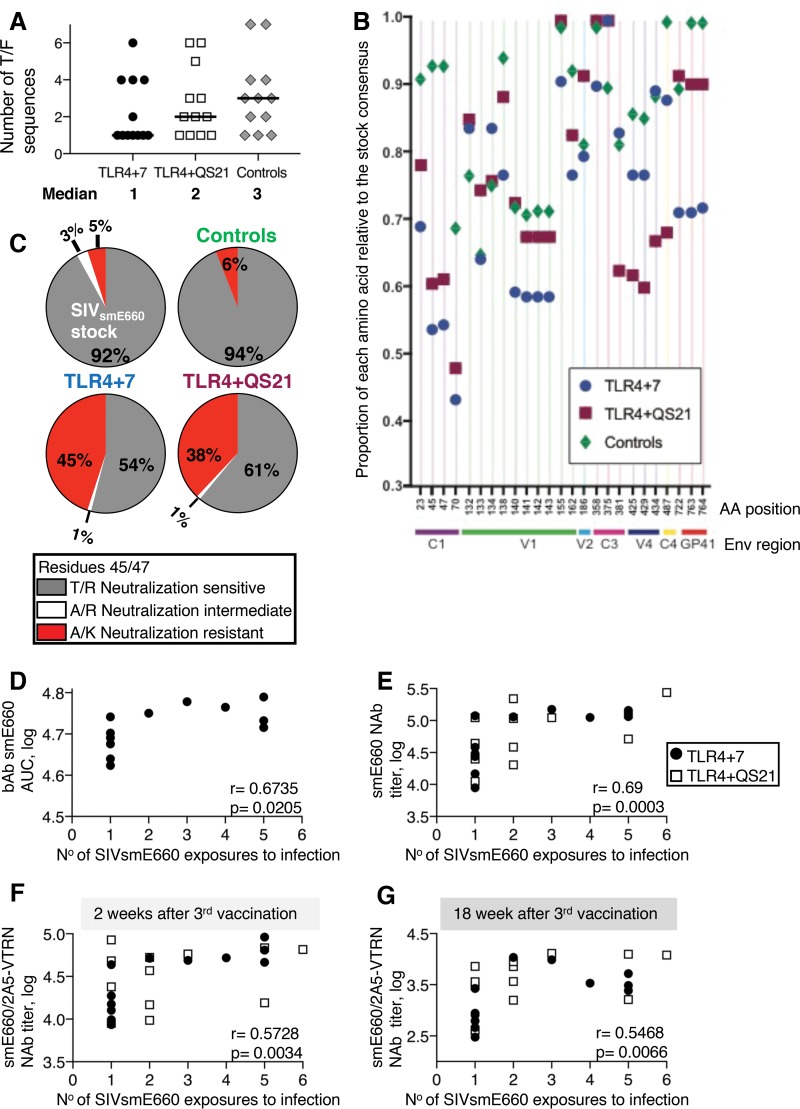
Sieve effect with selection of SIV_smE660_ neutralization-resistant virus variants. (A) Single-genome amplification and direct sequencing of the T/F *env* genes from the plasma samples (Table 1) were used to determine the number of T/F variants in the vaccinees and controls. (B) Genetic analysis of T/F Env sequences. Fifty-five informative sites were plotted as a proportion of each amino acid (AA) compared to the consensus sequence (see Fig. S6 in the supplemental material), and the plot shown excludes the 25 sites with a <10% difference in relative proportions between any of the groups for clarity. Residue 45 (T) and residue 47 (R), associated with neutralization resistance (54), showed the most prominent changes in the vaccinees compared to controls. (C) The percentage of Env sequences with consensus T/R (neutralization-sensitive), A/K (neutralization-resistant), and A/R (neutralization-intermediate) sequences are shown for the SIV_smE660_ challenge stock and the infected controls and vaccine groups. No viruses with T/K changes were found. (D to G) Two-tailed nonparametric Spearman correlation plots show direct correlations of SIV_smE660_ bAb in plasma of the TLR4+7 group (D), NAb to pseudotyped T/F SIV_smE660_-CG7G (E), the neutralization-sensitive SIV_smE660_ (SIVsmE660/2A5-VTRN) (F), and the number of SIV_smE660_ exposures to infection (G). Associations with bAb and NAb were measured 2 weeks after the 3rd vaccination (D to F) and 2 weeks before challenge (F). Spearman *r* and *P* values are shown.

Enumeration of the T/F viruses in the control group showed a range of 1 to 7 T/F variants (median of 3 T/F variants), indicating that the challenge virus inoculum contained more than one animal infectious dose (AID) (Fig. 4A; detailed in Table 1). Interestingly, measurements in the vaccine groups showed a lower number of T/F variants, with medians of 1 T/F variant for the macaques in the TLR4+7 group and 2 T/F variants for the macaques in the TLR4+QS21 group. Although the difference compared to the control group (median of 3) did not reach statistical significance, the TLR4+7 group showed a trend toward a lower number of T/F variants, with 7 of the 12 animals being infected with a single T/F variant compared to the control group, where only 3 of 12 animals were infected with a single T/F variant. These data suggest a sieve effect in the vaccinees, especially in the TLR4+7 group.

**TABLE 1:** Infection of vaccinees by neutralization-resistant SIV_smE660_ A/K variants

Group	Animal	TRIM-5α genotype	No. of exposures to infection	No. of Env proteins sequenced	% of Env proteins with nonconsensus A/K	No. of T/F variants	No. of T/F variants with A/K
TLR4+7	T065	R	4	13	100	1	1
TLR4+7	T066	M/S	1	13	85	6	4
TLR4+7	T069	M/S	2	10		2	
TLR4+7	T071	M/S	1	10		1	
TLR4+7	T074	M/S	1	13	100	1	1
TLR4+7	T075	R	1	9		4	
TLR4+7	T078	M/S	1	14	21	4	1
TLR4+7	T092	R	5	11		4	
TLR4+7	T093	R	5	14	100	1	1
TLR4+7	T094	M/S	1	12		1	
TLR4+7	T098	R	5	11	100	1	1
TLR4+7	T099	M/S	3	14		1	
TLR4+QS21	T072	M/S	1	14	100	1	1
TLR4+QS21	T076	R	3	14		1	
TLR4+QS21	T077	M/S	2	14		6	
TLR4+QS21	T079	M/S	1	11		1	
TLR4+QS21	T081	R	6	13	46	2	1
TLR4+QS21	T083	M/S	2	13		3	
TLR4+QS21	T084	M/S	1	13		5	
TLR4+QS21	T085	M/S	2	13	92	6	5
TLR4+QS21	T086	M/S	1	14	100	3	1
TLR4+QS21	T088	R	2	15	100	2	1
TLR4+QS21	T090	R	5	13		2	
TLR4+QS21	T091	R	5	12		1	
Control/TLR4+7	T080	R	4	14		2	
Control/TLR4+7	T082	M/S	2	13		1	
Control/TLR4+7	T095	R	1	11		7	
Control/TLR4+QS21	T073	M/S	1	11	18	4	1
Control/TLR4+QS21	T087	M/S	5	11		4	
Control/TLR4+QS21	T096	M/S	1	15		1	
Control naive	T067	M/S	1	16		3	
Control naive	T068	R	1	15		1	
Control naive	T070	R	1	10	20	7	1
Control naive	T089	M/S	1	12	42	3	1
Control naive	T097	M/S	1	11		3	
Control naive	T100	R	1	15	7	2	1

Analysis of the 457 Env amino acid sequences from the infected animals revealed changes in 54 informative positions (see Fig. S6 in the supplemental material; an excerpt of these data is shown in Fig. 4B), with more than one T/F Env having changes at each site. Comparison of sequences from the T/F variants in vaccinees to those in controls revealed several amino acid changes, with the most prominent changes at residue 23 (V), residue 45 (T), and residue 47 (R) in conserved region 1 (C1), with a 6- to 7-fold enrichment of such sequences in the vaccinees. Changes in the SIV_smE660_ consensus VTRS motif (residue 23 [V], residue 45 [T], residue 47 [R], and residue 70 [S]) to IAKN was previously reported as a hallmark characterizing the transmission of neutralization-resistant SIV_smE660_ variants (54). Of the 4 residues, changes in residues 45 and 47 from T/R to A/K were most strongly associated with the neutralization-resistant phenotype (54). This prompted a comparison of the virus variants in our challenge stock and the T/F variants in the infected animals (vaccinees and controls). We interrogated changes of the neutralization-sensitive T/R variant to the those of neutralization-resistant A/K variants, including A/R and T/K intermediates (Fig. 4C). This analysis showed that the SIV_smE660_ challenge stock used in this study contained ∼92% consensus T/R sequences, ∼3% intermediate A/R sequences, and 5% nonconsensus A/K sequences (Fig. 4C). The residue 23 V-to-I change was mostly found together with the T/R-to-A/K changes, while the residue 23 V-to-N change existed independent of A/K changes (Fig. 4B and Fig. S6). The T/F viruses in the 12 control animals showed a similar distribution of consensus T/R (∼94%) and nonconsensus A/K (6%) sequences as that of the challenge stock. Similar data were obtained in another 8 control animals challenged in a previous study with the same SIV_smE660_ stock (4% of T/F variants with nonconsensus A/K) (7). Interestingly, we found that animals in both vaccine groups showed a great enrichment of the nonconsensus neutralization-resistant A/K variant (Fig. 4C), representing 38 to 45% of the T/F sequences. Neutralization-resistant A/K viruses were the dominant T/F variants in 10 of 11 vaccinees (Table 1). In contrast, in control animals infected with the A/K variant, this virus was one of several T/F variants, never reaching more than 50% of the variants in plasma (Table 1). These data support a strong sieve effect with a skewing to infection by the nonconsensus A/K viruses in the vaccinated animals, suggesting a contribution of immune mechanisms, i.e., NAb, able to prevent or delay infection by the neutralization-sensitive T/R virus variants.

### Vaccine-induced SIV_smE660_-specific NAb responses contribute to a delay in SIV_smE660_ acquisition.

Interrogating the role of humoral immune responses to the delay in virus acquisition, we found that bAb to SIV_smE660_, but not to SIV_mac251_, in the TLR4+7 group showed a direct correlation with virus acquisition (Fig. 4D) (*P* = 0.0205). Furthermore, considering all the vaccinated animals, plasma NAb to SIV_smE660_-CG7G directly correlated with a delay in virus acquisition (Spearman *r* = 0.69; *P* = 0.0003) (Fig. 4E). In addition, we found a direct correlation of NAb to neutralization-sensitive SIV_smE660_/2A5-VTRN (tier 1) measured at the peak of viremia (week 2 after the 3rd vaccination) (Fig. 4F) and before challenge (week 18 after the 3rd vaccination) (Fig. 4G) and a delay in virus acquisition (Spearman *r* = 0.5728 and *P* = 0.0034, and Spearman *r* = 0.5468 and *P* = 0.0066, respectively). These data are in accord with the observed sieve effect (Fig. 4C) and showed that SIV_smE660_-specific NAb played a role in reducing the risk of infection (see Table S3 in the supplemental material). While these NAb delayed infection by neutralization-sensitive T/R viruses, the lack of NAb to the neutralization-resistant A/K variant resulted in increased infection by these variants (Fig. 4C).

The contribution of the vaccine-induced NAb to the delayed viral acquisition shown in Fig. 4E to G could not be established for the individual vaccine formulations because statistical analysis showed wide confidence intervals with only 12 animals per group. Nevertheless, we noted that the four outcomes in Fig. 4D to G are strongly correlated with each other in the TLR4+7 group, with the six pairwise correlation coefficients being between 0.73 and 0.87, whereas in the TLR4+QS21 group, the range is 0.22 to 0.69. Together, these data support the conclusion that the vaccine-induced antibody (SIV_smE660_-specific bAb and NAb) provided partial protection, contributing to the delay in infection upon repeated virus exposures.

### Contribution of humoral immune responses to control of viremia.

**FIG 5:**
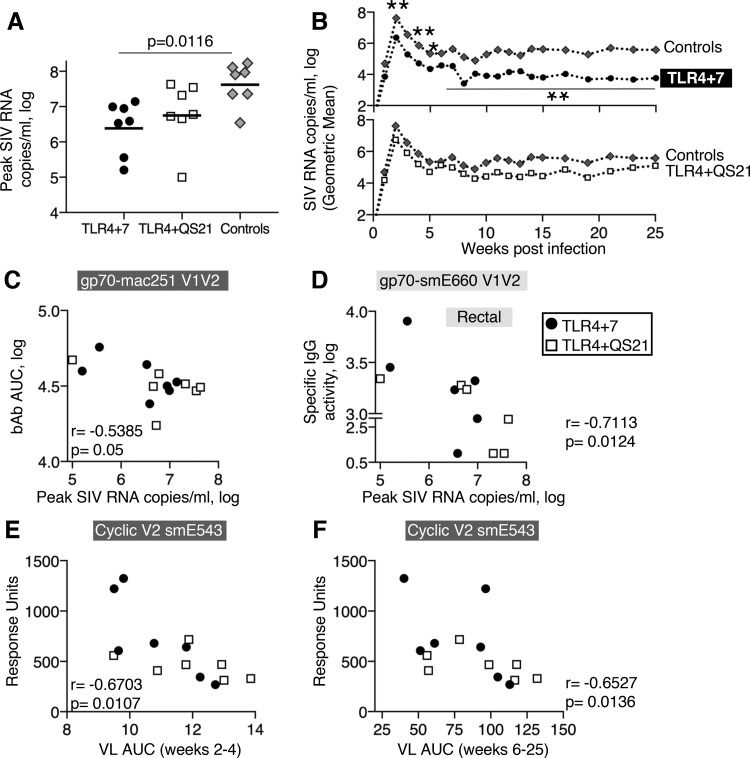
Control of viremia. (A) The dot plot shows the peak VL of each animal with the TRIM-5α M/S genotype, and the median is indicated. The *P* value is from multiple comparisons to controls using ANOVA (Kruskal-Wallis test). (B) Geometric means of virus loads monitored for 25 weeks are shown for TLR4+7 (*n* = 7) and controls (*n* = 7) (top) and TLR4+QS21 (*n* = 7) and controls (*n* = 7) (bottom). (C and D) Inverse correlation of SIV_smE660_-specific V1V2 responses in plasma (C) and rectal mucosa (D) and peak virus loads. gp70-V1V2 responses were measured by SIV-BAMA. (E and F) Inverse correlation of SIV_smE660_-specific cyclic V2 responses in plasma and virus loads (AUC) during the acute phase (weeks 2 to 4) (E) and chronic phase (weeks 6 to 25) (F) of infection.

To evaluate vaccine effects on the control of viremia, plasma SIV RNA virus loads (VL) were measured for 25 weeks postinfection (see Fig. S7 in the supplemental material). We observed significantly lower peak and chronic viremia in animals with the TRIM-5α R genotype than in animals with the TRIM-5α M/S genotype within the control group (Fig. S7), as reported by others (54). To evaluate potential vaccine effects on the postacquisition control of viral replication, independent of the effects of the restrictive TRIM-5α genotype, all subsequent postinfection analyses were performed using the subset of animals with the M/S genotype (Fig. 5). Of note, one animal (T073; TRIM-5α M genotype) of the control group had the highest VL and did not reach the study endpoint because it had to be sacrificed at week 18 due to AIDS-related disease (Fig. S7). Median peak VL of the vaccinees, measured at week 2 postinfection, were 1.3 logs (TLR4+7) and 1.1 logs (TLR4+QS21) lower than those of the controls and reached significance in the TLR4+7 group (*P* = 0.0116) (Fig. 5A). Analysis of VL postpeak in the TLR4+7 group showed significantly lower viremia during the early phase at weeks 2, 3, and 4 (*P* = 0.014, 0.019, and 0.029, respectively) and during chronic infection at weeks 6 to 25 (*P* = 0.026), demonstrating durable lower viremia in this vaccine group (Fig. 5B). Peak VL directly correlated with the acute phase (area under the curve [AUC], 2 to 4; *n* = 36; *r* = 0.947; *P* < 0.0001) and chronic phase (AUC, 6 to 24; *r* = 0.7583; *P* < 0.0001).

To assess the contribution of vaccine-induced humoral responses to the control of viral propagation, we analyzed antibody responses in relation to viremia. We found inverse correlations of plasma and mucosal V1V2 responses and peak VL (Fig. 5C and D, respectively). Cyclic V2 responses inversely correlated with postpeak and chronic viremia (Fig. 5E and F, respectively). Interestingly, both plasma and mucosal humoral immune responses, including V2-specific responses, were associated with delayed virus acquisition (Fig. 4D to G) and postinfection control (Fig. 5 and Table S3).

### SIV-specific T cells in both vaccine groups contribute to control of viremia.

**FIG 6:**
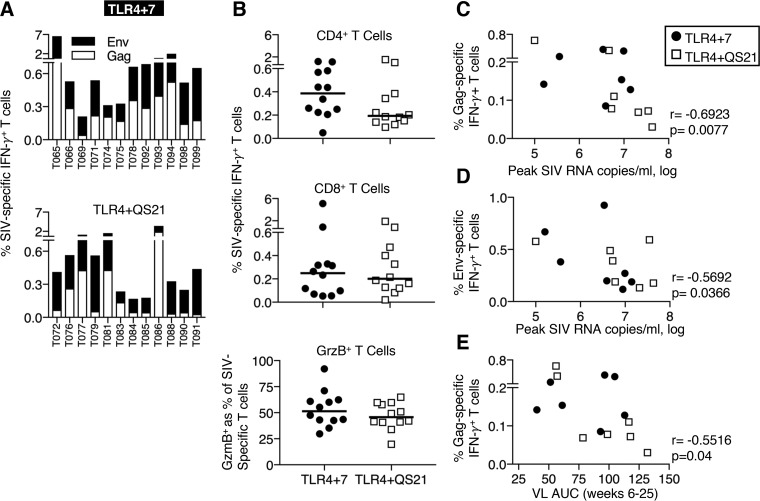
Antigen-specific T cell responses and control of viremia. (A) Bars show frequencies of Env-specific and Gag-specific T cell responses measured in PBMC samples collected 2 weeks after the 3rd vaccination. (B) Dot plots show SIV-specific IFN-γ^+^ CD4^+^ (top), CD8^+^ (middle), and granzyme B-positive (GrzB^+^) (bottom) SIV-specific T cells. (C to E) Inverse correlations of Gag-specific IFN-γ^+^ T cells (C) and Env-specific IFN-γ^+^ T cells (D) measured 2 weeks before challenge start and virus load at peak and between Gag-specific IFN-γ^+^ T cells measured 18 weeks after the 3rd vaccination and VL during the chronic phase (weeks 6 to 25 postinfection) (E). Spearman *r* and *P* values are shown.

We performed a detailed analysis of vaccine-induced T cell responses, measuring peak responses (week 2 after the 2nd and 3rd vaccinations), durability of responses (18 weeks after the 3rd vaccination), and anamnestic responses after infection (weeks 4 and 8). Animals in both vaccine groups developed robust Env- and Gag-specific T cell responses ranging from 0.2 to 7% of total T cells in blood (Fig. 6A), with the variability expected among outbred animals. Comparison of the antigen-specific IFN-γ^+^ T cell subsets between the groups showed similar levels of CD4^+^ (Fig. 6B, top) and CD8^+^ (Fig. 6B, middle) T cells, cytotoxic potential (GrzB^+^) (Fig. 6B, bottom), and durability over 4 months after the last vaccination (see Fig. S4C in the supplemental material).

We next investigated the contribution of the SIV-specific T cell responses to virus control. The association of Gag and Env T cell responses (measured 2 and 18 weeks after the 3rd vaccination) with peak VL and viremia during the chronic phase was determined (Table S4). Specifically, we found significant inverse correlations of vaccine-induced Gag-specific IFN-γ^+^ T cell responses, measured 18 weeks after the 3rd vaccination (2 weeks before the start of repeated virus exposures), with peak (Fig. 6C) and chronic (Fig. 6E) viremia and of Env-specific T cell responses with peak VL (Fig. 6D). In addition, detailed analysis of antigen-specific T cell subsets measured 2 weeks before challenge (week 18 after the 3rd vaccination) (Fig. S8A and S8B) also indicated a significant correlation with the reduction of peak, postpeak, and chronic viremia (Table S4).

Comparison of Gag- and Env-specific T cells 2 weeks before challenge and at 8 weeks postinfection (Fig. S8A and S8B) showed robust anamnestic T cell responses with a significant increase in the frequency of the SIV Gag (*P* = 0.003)- and Env (*P* = 0.009)-specific CD8^+^ T cell subset (Wilcoxon matched-pairs signed-rank test). Both vaccine groups also showed a significant increase in the frequency of the SIV-specific memory T cell subset with cytotoxic potential (CD95^+^ CD28^−^ GrzB^+^) (Fig. S8C) (*P* = 0.007 and *P* = 0.005, respectively) upon infection. Interestingly, this CD8^+^ T cell subset inversely correlated with early viral control, including peak and postpeak viremia (weeks 2 to 4) (Fig. S8D, top and bottom, respectively). Together, these data support our previous findings (6, 7), showing that DNA and DNA-protein vaccination induced potent T cell responses able to contribute to the containment of the infection.

**FIG 7:**
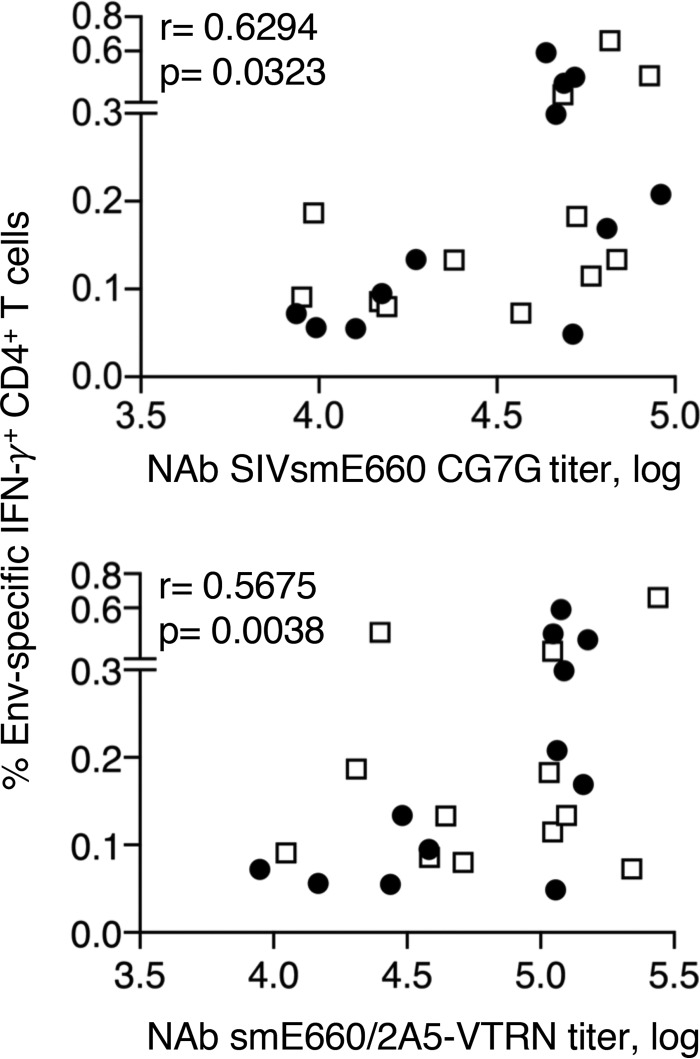
Association between Env-specific CD4^+^ T cells and NAb. Shown are direct correlations of Env-specific CD4^+^ T cells measured 2 weeks after the 3rd vaccination and NAb to SIV_smE660_ (SIV_smE660_-BR-CG7G [left] and SIV_smE660_/2A5-VTRN [right]) in plasma. Spearman *r* and *P* values are shown.

In addition to their role in the control of viremia, we further found an association of Env-specific T cells with humoral immune responses. In particular, we found direct correlations of Env-specific CD4^+^ T cells with NAb against SIV_smE660_-CG7G and SIV_smE660_/2A5-VTRN (Fig. 7), NAb responses which were also found to be correlates of a delay in infection (Fig. 4E to G). These data support a helper function of vaccine-induced Env-specific CD4^+^ T cells in humoral immune response development. Together, our data show that vaccine-induced humoral and cellular immune responses contribute to the delay of virus acquisition and control of viremia, and they further revealed a connection of the two arms of vaccine-induced immunity.

## DISCUSSION

This study used two adjuvant formulations with the same DNA-protein combination vaccine. Comparison of humoral and cellular immune responses showed that both vaccines induced robust responses of similar magnitudes but also showed qualitative differences between the vaccine groups. Despite these differences, none of those features could be linked to better protective responses between the two vaccine groups. TRIM-5α R animals in both groups showed a decreased risk of virus acquisition due to the vaccine, in agreement with data from other reports (31, 54–56). We found that both TRIM-5α R and M/S animals with mucosal V1V2 Ab showed a significantly decreased risk of virus acquisition due to the vaccine, and the vaccine effect was stronger in the TRIM-5α R animals. A sieve effect was found for animals in both groups, an indication that the NAb produced by the vaccines are protective. The TLR4+7-adjuvanted vaccine induced humoral responses with effector functions, including ADCC, in more vaccinees and broader NAb. In addition, TRIM-5α M/S animals in TLR4+7 group showed reduced peak viremia.

This study is an extension of our previous work using a DNA-protein coimmunization regimen in rhesus macaques using a SIV_mac251_-derived vaccine and heterologous SIV_smE660_ challenge. Whereas in a previous study, we used AT-2-inactivated virus particles as the protein immunogen (7), in the present study, we tested this vaccine concept using purified SIV gp120 formulated with either of two TLR4 agonist-based adjuvants, TLR4+7 and TLR4+QS21. In a pilot study, we had shown that an HIV DNA-protein vaccine using TLR4 (GLA-SE)-adjuvanted HIV gp120 induced higher systemic antibody responses than DNA alone in rhesus macaques (13). A SIV DNA-protein vaccine using adjuvanted Env also showed improved mucosal antibody dissemination compared to a DNA-only vaccine (14). We tested the efficacy of such SIV DNA-protein vaccine coimmunization regimens upon heterologous challenge administered 5 months after the last vaccination.

We found a strong correlation of V2-specific responses, including mucosal V1V2 and cyclic V2, with control of viremia. A role of V2-specific immune responses as a correlate of protection was first identified in the RV144 trial (1–4) and subsequently also reported for different vaccine-challenge models in macaques (70, 80–83). In addition to humoral responses, the DNA-based vaccines induced cellular responses associated with reductions of peak and chronic viremia, in agreement with our previous data (5–8). Interestingly, we found a positive association of Env-specific CD4^+^ T cells and NAb, a correlate of protection. Thus, these data suggest a role of Env-specific CD4^+^ T cells in providing help to B cells, thereby contributing to antibody development (84, 85) and resulting in the development of stronger protective immune responses.

Our data show that the two vaccine formulations differentially affected antibody features and functions, pointing to the importance of adjuvant selection. Others reported that comparing alum and M59 adjuvants revealed distinct responses and virological outcomes (82), and comparing alum-TLR7 and MF59 revealed the induction of differential innate profiles (41). Others have used the TLR4-TLR7 adjuvant combination together with SIV/HIV Env and found an important role of the formulation (alum versus adjuvant nanoemulsion) in affecting innate responses (26) and robust immunogenicity in mice and macaques (26, 31, 41, 46). In particular, Iyer et al. (46) reported that a DNA/modified vaccinia virus Ankara (MVA) vaccine followed by virus-like particles (VLPs) formulated in the TLR4/7/8 adjuvant combination increased humoral immune responses and protection from SIV_mac251_ challenge. Kasturi et al. (31) recently reported that a nanoparticle-formulated vaccine containing a combination of TLR4 and TLR7/8 agonists together with soluble recombinant SIV_mac239_-derived Env gp140 and Gag protein or with VLPs containing SIV_mac239_ Env and Gag induced persistently high antibody responses that were able to control SIV_smE660_ challenge in macaques carrying TRIM-5-α-restrictive alleles. Here, we found that vaccines formulated with both TLR4+7 and TLR4+QS21 adjuvants induced robust and durable antibody responses that efficiently disseminated to mucosal surfaces and delayed SIV_smE660_ acquisition in macaques carrying TRIM5-α-restrictive alleles, and in addition, the vaccine-induced humoral and cellular immune responses help to control acute and chronic viremia in immunized animals. Thus, as noted by others, the TRIM-5α-resistant allele influences the rate of viral acquisition (50–55), and this effect is increased in vaccinated macaques (31, 41, 46, 54–56). In agreement with those reports, we also found that the TRIM-5α genotype acts as a confounding contributor, together with cross-clade SIV_smE660_-specific NAb and mucosal V1V2 bAb, to vaccine-induced control of SIV_smE660_ acquisition. Thus, the TRIM-5α R genotype as an innate protective immune mechanism can assist vaccine-induced immune responses in controlling infection. Here, we show that animals of both the TRIM-5α R and M/S genotypes benefit from the vaccine, but the TRIM-5α R animals showed the strongest protection from SIV_smE660_ acquisition. We hypothesize that the observed protective efficacy could have been stronger using a lower inoculation dose since our controls were infected with a median of 3 T/F variants.

The SIV_smE660_ stock swarm used as the challenge stock in this work offered an additional opportunity to explore vaccine efficacy since this swarm comprises neutralization-sensitive and -resistant viruses. This viral diversity allowed monitoring of the effectiveness of the vaccine-induced responses against viruses with different neutralization properties. Indeed, we found that both vaccine groups showed a significant sieve effect, with ∼50% of the animals preferentially infected by T/F variants with the neutralization-resistant A/K genotype. This represents an ∼10-fold enrichment compared to the low fraction in the stock (∼5%) and in infected control animals and clearly points to vaccine-induced immune responses that are able to delay or block infection. Using a DNA-adenovirus type 5 (Ad5) vaccine, protection against a neutralization-sensitive T/R variant and a sieve effect resulting in a significant overrepresentation of neutralization-resistant A/K SIV_smE660_ were reported (54). Using an Ad prime-Env protein boost vaccine, protection against the neutralization-sensitive T/R SIV_smE660_ was recently reported; however, due to the low number of infected animals, no clear sieve effect could be established (55). Thus, the study presented here comparing T/F variants of vaccinees and controls and other studies (54) clearly showed that A/K virus selection is linked to a vaccine-induced protective mechanism and not to innate selection, since the controls and the challenge stock show the same frequency of A/K virus variants. Also, we and Roederer et al. (54) did not observe selection exerted by the mucosal route of infection, as reported by others (86). One important consideration is that SIV_smE660_ swarms used by different laboratories differ in the compositions of viral quasispecies with specific and known neutralization properties (53). It was further noted by others that the A/K mutation at residues 45 and 47 may not be the only decisive factor to render a virus neutralization resistant (42). This is also exemplified by our finding of robust NAb against SIV_smE660_ T/F Env CG7V (Fig. 4E), a tier 1 Env that contains the A/K mutations. Collectively, these studies show that the SIV_smE660_ swarm is a useful challenge virus that allows the exploration of vaccine efficacy in relation to specific protective responses by testing specific neutralization variants.

Different nonhuman primate (NHP) models are being used for AIDS pathogenesis and vaccine studies using different challenge viruses, and each model is associated with advantages and disadvantages (reviewed in reference 87). In this study, we used Indian rhesus macaques vaccinated with a SIV_mac251_-derived DNA-protein vaccine and challenged by the heterologous SIV_smE660_. This model was selected because the SIV_smE660_ challenge stock comprises more neutralization-sensitive virus variants than SIV_mac251_ (53–55), providing an advantage for testing antibody-mediated protection. However, SIV_smE660_ is susceptible to restriction by the macaque TRIM-5α alleles, a cross-species restriction factor (47–53). Thus, our analysis took into consideration the putative impact of TRIM-5α on virus acquisition and vaccine efficacy. In addition to the vaccine effect on TRIM-5α R animals, the data also support a significant vaccine effect, as shown in Fig. 3C, where the TRIM-5α M/S animals that developed V1V2 mucosal responses showed a reduced risk of virus acquisition (*P* = 0.028). Therefore, the relevance of these results is not restricted to TRIM-5α R animals only.

In conclusion, these data show that a combination of genetic makeup (TRIM-5α R genotype) and vaccine-induced immune responses against SIV_smE660_, in particular SIV_smE660_-specific NAb, skewed infection toward the neutralization-resistant A/K variants. Importantly, mucosal SIV_smE660_-specific V1V2 responses also contributed significantly to reduced susceptibility to SIV_smE660_ infection in both the TRIM-5α R and M/S groups, and in addition, both V2-specific antibodies and cellular responses contributed to the control of viremia. In summary, TLR4-based adjuvants included in the DNA-protein combination vaccine induced immune responses associated with a delay of virus acquisition and control of viremia.

## MATERIALS AND METHODS

### Ethics statement.

All animals were cared for and procedures were performed under a protocol approved by the Institutional Animal Care and Use Committee of Bioqual, Inc. (animal welfare assurance no. A3086-01; protocol no. 15-008), and the USDA (certificate no. 51-R0036). The macaques in this study were managed according to the animal husbandry program, which aims at providing consistent and excellent care to nonhuman primates at the vivarium. This program operates based on the laws, regulations, and guidelines promulgated by the U.S. Department of Agriculture (e.g., the Animal Welfare Act and its regulations and the Animal Care Policy Manual), the Institute for Laboratory Animal Research (e.g., *Guide for the Care and Use of Laboratory Animals*, 8th ed. [88]), the Public Health Service, the National Research Council, the Centers for Disease Control and Prevention, and the Association for Assessment and Accreditation of Laboratory Animal Care (AAALAC) International. The nutritional plan utilized by Bioqual, Inc., consisted of twice-daily feeding of Labdiet 5045 high-protein primate diet, and food intake was closely monitored by animal research technicians. This diet was also supplemented with a variety of fruits and vegetables as part of the environmental enrichment program established by the veterinary staff and enrichment technician. Pairing of animals as part of the environmental enrichment program was managed by the enrichment technician. All primary enclosures and animal rooms were cleaned daily with water and sanitized at least once every 2 weeks. Macaques (*n* = 36) used in this study were 24 males and 12 females. Their median weight was 3.6 kg, and their median age was 2.9 years. The animals were negative for the Mamu A*01, B*08, B*17, and B*29 alleles and for Simian T cell leukemia virus (STLV) (PCR/seronegative) and were screened for TRIM-5α genotypes. The three balanced cohorts of animals (*n* = 12/group) were grouped according to sex and TRIM-5α genotype, as described in Table S1 in the supplemental material. Vaccinations were performed under anesthesia (ketamine administered at 10 mg/kg of body weight), and all efforts were made to minimize suffering. No adverse effects were found. All animals were euthanized at the end of the study.

### Plasmid DNA and *in vitro* transfection.

The DNA vaccination included plasmids expressing soluble trimeric gp140 Env from SIV_mac239_ (plasmid 237S) and SIV_mac251_ T/F M766 (plasmid 241S) (64) and plasmids expressing the membrane-bound gp120e-TM proteins (plasmid 266S and plasmid 267S). SIV_mac239_ gp120e-TM (plasmid 266S) and SIV M766 gp120e-TM (plasmid 267S) consist of the gp120 region with an additional 54 C-terminal amino acids spanning the fusion peptide and part of the heptad region fused with the transmembrane (TM) domain. gp120-TM consists of M766 gp120 fused to the TM domain (plasmid 268S). All vaccine plasmids comprised the eukaryotic expression vector pCMV.Kan (12).

For transient expression, the plasmids (200 ng) were transiently transfected into HEK293 cells (64), and the supernatant and cells were collected 2 days later. The cells were harvested in 1 ml of N1 buffer (20 mM HEPES, 10% glycerol, 1 mM MgCl_2_, 400 mM NaCl, 0.5% Triton X-100, and 1 tablet of a protease inhibitor cocktail [Roche]) and sonicated with two 6-s bursts on ice. Extracellular and cell-associated fractions (1/200 of each fraction) were loaded onto a 10% NuPAGE gel and transferred to nitrocellulose membranes. Western immunoblot analysis was performed using a mouse anti-SIV gp120 Ab (1:5,000 dilution) (catalogue no. 1487; AIDS Reagent Program), followed by horseradish peroxidase (HRP)-labeled sheep anti-mouse IgG (1:10,000 dilution) (GE Healthcare UK). As a loading control, the cell-associated fraction was probed with antiactin antibody (1:10,000 dilution) (clone C4; EMD Millipore, Billerica, MA). The bands were visualized using the enhanced chemiluminescence (ECL) Western blotting detection system (GE HealthCare). Images of the blots were acquired by using a ChemiDoc XRS^+^ imager and Bio-Rad imageLab (Bio-Rad).

The cell membrane localization of gp120e-TM was evaluated by surface staining with antibodies targeting Env, followed by flow cytometry. Briefly, 100 ng of the two membrane-bound gp120e-TM plasmids (mac239 and M766) was transiently transfected into HEK293 cells. The next day, the cells were harvested, washed with phosphate-buffered saline (PBS)–0.2% heat-inactivated human serum, and incubated with 2 μg of anti-gp120 (catalogue no. 1487; AIDS Reagent Program), followed by the addition of allophycocyanin (APC)-labeled goat anti-mouse IgG. After washing the unbound antibody, data were acquired in a Fortessa flow cytometer (BD Biosciences, San Jose, CA), and the data were analyzed using the FlowJo software platform (Tree Star, Inc., Ashland, OR).

### Vaccination and challenge.

Two groups of animals (*n* = 12) were coimmunized with DNA and the gp120 Env protein. Each animal received two DNA vaccine mixtures (with mac239 and M766 Env, respectively) delivered via intramuscular (i.m.) injection in the left and right inner thighs followed by *in vivo* electroporation (EP) using the Elgen 1000 device (Inovio Pharmaceuticals, Inc., Plymouth, PA). The adjuvanted recombinant protein was administered by needle and syringe at the same anatomical location immediately following DNA EP.

The two SIV DNA mixtures (1.1 mg total DNA each) for the 1st and 2nd vaccinations contained a mixture of (i) 0.5 mg Gag DNA plasmids (p57gag [plasmid 206S] and MCP3gag [209S]) and 0.5 mg of mac239 *env* DNA plasmids (gp140 [237S] and gp120e-TM [266S]) (left side administration) and (ii) 0.5 mg Gag DNA and 0.5 mg of M766 *env* DNA plasmids (gp140 [241S] and gp120e-TM [267S]) (right side administration). The DNA dose for the 3rd vaccination (2.1 mg total DNA each) consisted of 1 mg of *env* and *gag* DNA. The *gag* DNA mixture for the 3rd vaccination included 0.5 mg p27CE1 and p27CE2 conserved element DNA (262S and 263S) (89). All DNA formulations, including the sham DNA, contained 0.1 mg of rmIL-12 DNA (plasmid AG157) per injection.

The recombinant SIV_mac251_ M766 Env gp120 protein (200 μg) was formulated in PBS with TLR4+7 (LS144 [10 μg GLA and 50 μg imiquimod in liposomes]) or TLR4+QS21 (LS131 [10 μg GLA-LSQ liposomes]), obtained from the Infectious Disease Research Institute (IDRI), Seattle, WA. Six control animals received 2 mg (months 0 and 2) or 4 mg (month 6) of sham DNA (plasmid CMVkan) by EP. For the 3rd sham vaccination, the animals also received 10 μg of TLR4+7 (*n* = 3) and TLR4+QS21 (*n* = 3), respectively. Six control animals were treatment naive.

The animals were challenged six times by the intrarectal route with weekly exposures using a 1:50 dilution of a SIV_smE660_ stock, as previously reported (7, 76). To assess take of infection and potential postinfection viral control, plasma SIV RNA levels were measured using a quantitative real-time PCR (qRT-PCR) assay (90) with a detection limit of 15 viral RNA copies/ml. To evaluate the number of distinct transmitted variants, single-genome amplification was performed at the peak of viral replication (2 to 3 weeks postinfection) (55, 79).

### Humoral responses and functional assays.

Pepscan analyses were performed by an enzyme-linked immunosorbent assay (ELISA) at a dilution of 1:50 using 115 peptides (20-mer overlapping by 14 aa) spanning SIV_mac251_ Env from aa 16 to 717 (amino acid numbering according to SIV_mac239_) (Advanced Bioscience Lab, Rockville, MD). Binding antibody to SIV_mac251_ Env gp120 and p27^Gag^ was measured using serial dilutions of plasma tested by a standard ELISA (Advanced Bioscience Lab, Rockville, MD). Endpoint binding titers are reported as the reciprocal of the highest dilution scoring positive (having a value higher than average values obtained with naive macaque plasma plus 2 standard deviations). Concentrations of SIV-specific IgG to SIV_mac251_ gp130, SIV_smE660_-CG7V gp140 Env, gp70-SIV_mac251_ V1V2, and gp70-SIV_smE660_ V1V2 in plasma rectal and vaginal mucosa were measured by SIV binding antibody multiplex assay (SIV-BAMA) using a custom SIV multiplex ELISA (7, 91–93). Briefly, SIV Env and V1V2 antigens were coupled to carboxylated fluorescent beads and incubated with diluted test samples. SIV-specific IgG were detected with biotinylated goat anti-monkey IgG, followed by incubation with streptavidin-phycoerythrin (PE). Beads were washed, and data were acquired on a Bio-Plex instrument to measure florescence intensity. Plasma samples were tested in serial dilutions, and the area under the mean fluorescence intensity (MFI)-plasma dilution curve (AUC), calculated using the trapezoidal curve fit method, was reported for plasma samples. Mucosal samples were tested at a 1:2 dilution. Specific binding activity values, calculated as MFI × dilution/total IgG concentration (micrograms per milliliter), are reported. The total IgG concentration in mucosal samples was measured by a custom ELISA after sample elution and preparation for binding antibody assays. The SIV-BAMA was run under good clinica laboratory practice (GCLP)-compliant conditions, including tracking of positive controls by Levy-Jennings charts, using 21 CFR part 11-compliant software. The rectal and vaginal samples were assayed at a dilution of 1:2, and the binding magnitude is reported as specific activity (MFI × dilution/total IgG concentration, in micrograms per milliliter). Neutralizing antibody activity was measured in 96-well culture plates by using Tat-regulated luciferase (Luc) reporter gene expression to quantify reductions in virus infection in TZM-bl cells. TZM-bl cells were obtained from the NIH AIDS Research and Reference Reagent Program, contributed by John Kappes and Xiaoyun Wu (94–98). Assays were performed with SIV Env-pseudotyped viruses as described previously (99). Heat-inactivated (56°C for 30 min) serum samples were diluted over a range of 1:20 to 1:43,740 or 1:300 to 1:23,437,500 (for assays against tier 1A SIV_mac251.6_ and SIV_smE660_/2A5-VTRN pseudovirus) in cell culture medium and preincubated with virus (∼150,000 relative light unit equivalents) for 1 h at 37°C before the addition of cells. Following a 48-h incubation, cells were lysed, and Luc activity was determined using a microtiter plate luminometer and BriteLite Plus reagent (PerkinElmer). Neutralization titers are the sample dilution (for serum) or concentration in micrograms per milliliter at which relative luminescence units (RLU) were reduced by 50% compared to RLU in virus control wells after subtraction of background RLU in cell control wells.

Cyclic V2 responses were measured in plasma (1:40 dilution, in triplicate) by surface plasmon resonance (SPR; Biacore) using N-linked biotinylated cyclic SIV_mac251_ V2 and SIV_smE543_ V2 peptides captured onto streptavidin-immobilized CM7 sensor chips, followed by secondary IgG anti-monkey antibodies. The data were evaluated by using Biacore 4000 evaluation software 4.1 (82, 100). The SIV V2 peptide sequences used were as follows: CIAQNNCTGLEQEQMISCKFNMTGLKRDKTKEYNETWYSTDLVCEQGNSTDNESRCY [SIV_mac251_(F)], CKFNMTGLKRDKTKEYNETWYSTDLVSEQGNSTDNESRC [SIV_mac251_(S)], CIKNNSCAGLEQEPMIGCKFNMTGLKRDKKIEYNETWYSRDLICEQPANGESKCY [SIV_smE543_(F)], and CKFNMTGLKRDKKIEYNETWYSRDLISEQPANGSESKC [SIV_smE543_(S)]. ADCC activity was measured with the flow-based ADCC-GranToxiLux assay as described previously (77, 101), using CEM.NKRCCR5 target cells coated with SIV_mac251_ gp120 protein. Specific killing is defined as the percentage of gp120-coated target cells taking up granzyme B, with a positivity cutoff at 8%. The magnitude of ADCC responses was evaluated according to two parameters: (i) endpoint titers of plasma antibodies mediating ADCC and (ii) maximum percent GrzB activity. ADCC was also measured using SIV_mac251_- and SIV_smE660_-infected target cells according to previously reported methods (102).

### Systems serology assays and analysis.

Analysis of NK functions; ADCC, ADCD, antibody-dependent phagocytosis (ADCP), and antibody-dependent neutrophil phagocytosis (ADNP) effector functions; and antibody glycosylation was performed, as previously described (82, 103), using purified SIV_mac251_ M766 gp140 Env. Briefly, ADCP and ADNP of antigen-coated beads were performed using SIV_mac251_ M766 gp140-coated fluorescent beads. Beads were incubated with plasma from vaccinated animals, washed, and then placed in a coculture with THP1 cells or primary human neutrophils, respectively, as effector cells. The level of phagocytosis was quantified as the composite of the percentage and mean fluorescence intensity of bead uptake. Antibody-dependent NK cell activation was analyzed using SIV_mac251_ M766 gp140-coated 96-well plates. Plasma was added to antigen-coated plates, after which nonbinding antibodies were washed away, and purified human NK cells were added in the presence of brefeldin A. The levels of degranulation, interferon gamma (IFN-γ), and macrophage inflammatory protein 1β (MIP-1β) were measured. Antibody-dependent complement deposition was measured on beads using SIV_mac251_ M766 gp140-coated beads. Antigen-coated beads were cultured with plasma and washed to remove all nonbinding antibodies, and guinea pig complement was then added. The level of C3 deposition was then detected by flow cytometry. Finally, ADCC was measured using a rapid fluorescent cytotoxicity assay, whereby CEM cells were coated with SIV_mac251_ M766 gp140, after which the cells were washed and labeled with 2 distinct dyes to label the cellular membrane and cytoplasm. The targets were then incubated with primary human NK cells at an effector-to-target cell ratio of 1:10, and the level of death was measured by flow cytometry as the relative change in intact membrane/cytoplasm targets. All assays were repeated in duplicate, and noninfected NHP plasma background values were subtracted for each assay.

LASSO provided an unbiased and stringent variable selection technique to identify a minimal set of markers that best explain the differences between animals of each group. LASSO picked only individual markers from blocks of correlated variables, and the stringency in variable selection helps avoid overfitting. We then used PLSDA on the LASSO-selected features and obtained a reasonably good separation between the two groups. We validated the model in a rigorous 5-fold cross-validation framework and found that the actual model was significantly better than 2 null models.

### Measurement of SIV-specific T cells and flow cytometry.

SIV-specific T cell responses were measured by intracellular cytokine staining using peripheral blood mononuclear cells (PBMC) (0.6 × 10^6^ cells/sample) stimulated with Gag or Env (derived from M766 or CG7V) peptide pools (15-mer peptides overlapping by 11 aa) at a final concentration of 1 μg/ml for each peptide in the presence of monensin (Golgi-stop; BD Pharmingen, San Jose, CA). For negative and positive controls, PBMC were cultured in medium without peptides or stimulated with a phorbol 12-myristate 13-acetate (PMA) cell stimulation cocktail (eBioscience, Affymetrix, Inc., San Diego, CA, USA). After a 12-h incubation, cells were washed with PBS supplemented with 0.2% heat-inactivated human serum and surface stained as previously described (7, 15, 89, 104), using the following antibody mixes: CD3-allophycocyanin (APC)-Cy7 (clone SP34-2; BD Pharmingen, San Jose, CA), CD4-V500 (clone L200; BD Pharmingen), CD8-Alexa Fluor 405 (clone 3B5; Invitrogen, Carlsbad, CA), CD28-peridinin chlorophyll protein (PerCP) Cy5.5 (clone CD28.2; Bio-Legend, San Diego, CA), CD95-fluorescein isothiocyanate (FITC) (clone DX2; BD Pharmingen), and CCR7-APC (clone 150503; Invitrogen, Carlsbad, CA). Intracellular staining was performed after fixing and permeabilizing the cells in fixation/permeabilization buffer (eBioscience, Affymetrix, Inc., San Diego, CA) for 30 min at 4°C. After washing the cells with permeabilization buffer (eBioscience, Affymetrix, Inc.), the cells were stained with an antibody mix containing anti-IFN-γ–phycoerythrin (PE) Cy7 (clone B27; BD Pharmingen), anti-granzyme B-PE (clone GB12; Invitrogen), and anti-Ki67-Alexa Fluor 700 (clone B56; BD Bioscience) in permeabilization buffer. After 30 min of incubation at room temperature, the samples were washed and data were acquired on an LSR II or Fortessa flow cytometer (BD Biosciences, San Jose, CA). All the flow data were analyzed using FlowJo software (Tree Star, Inc., Ashland, OR). Samples were considered positive if the IFN-γ^+^ T cell frequency was at least 2-fold higher than the value for the medium control, and the numbers after subtraction of the values obtained for the negative controls were ≥0.05% of total T cells. For T cell phenotyping, cells were stained as described above, using the following antibodies: CD3-APC-Cy7, CD4-V500, CD8-Alexa Fluor 405, α4β7-APC (NHP Resource Reagents), CXCR3-PE-Cy7 (clone 1C6; BD Bioscience, San Jose, CA), HLA-DR–FITC (clone TU39; BD Bioscience, San Jose, CA), granzyme B-PE, and Ki67-Alexa Fluor 700. All the data were acquired and analyzed as described above.

### Single-genome amplification, sequencing, and T/F variant enumeration.

From each plasma specimen and the challenge inoculum, viral RNA was extracted using the QIAamp viral RNA minikit (Qiagen). RNA was eluted and immediately subjected to cDNA synthesis as previously described (55). The newly synthesized cDNA was serially diluted and distributed among 96-well plates so as to identify a dilution where PCR-positive wells constituted <30% of the total number of reactions. PCR amplification was performed as described previously (55). All PCR procedures were performed under PCR clean-room conditions using procedural safeguards against sample contamination. Correctly sized amplicons determined by electrophoresis on an agarose gel were directly sequenced by cycle sequencing using BigDye Terminator chemistry. Individual sequence fragments for each amplicon were assembled and edited using Sequencher (Gene Codes). All sequences were aligned and phylogenetic trees were constructed using ClustalW with manual editing in MacClade. Each low-diversity lineage was compared to a mathematical model of viral diversification over time to identify all transmitted/founder (T/F) lineages, as previously described (79). Each T/F variant from all infected animals as well as stock sequences were used to generate a consensus amino acid sequence. Each T/F lineage was then compared to the consensus and plotted as a fraction of the consensus for each vaccine group regardless of the challenge dose. In total, 54 sites were identified as being informative, excluding single sequence polymorphisms. Twenty-five sites showed a >10% proportional difference between groups.

### Statistical analyses.

Univariate statistical analyses were performed using Prism version 7 (GraphPad Software) or SAS. Comparisons were done using nonparametric *t* tests, as appropriate, and analysis of variance (ANOVA). Multivariate LASSO/PLSDA models were generated (72), and the accuracy of each of these models was measured using a 5-fold cross-validation setup. The animals were split into five subsets such that for each fold, four subsets were used for training, and the fifth one served as the test set. This process was repeated five times, i.e., across the 5 folds, with each subset serving as the test set once. For each fold, only the training samples for that fold were used for both LASSO-based feature selection and subsequent PLSDA-based classification. This entire procedure constitutes one “5-fold cross-validation run.” The median classification accuracy across 100 independent cross-validation runs was measured. Two different negative-control “null” models were defined based on permuted data as well as a random size-matched set of features.

## Supplementary Material

Supplemental material
