# Supplementary material for: Control of Heterologous Simian Immunodeficiency Virus SIVsmE660 Infection by DNA and Protein Coimmunization Regimens Combined with Different Toll-Like-Receptor-4-Based Adjuvants in Macaques
Source: J Virol. 2018 Jul 17;92(15):e00281-18. doi: 10.1128/JVI.00281-18 (PMC6052320; doi:10.1128/JVI.00281-18)
Supplement: Supplemental material [file supp_92_15_e00281-18__index.html]

Control of Heterologous Simian Immunodeficiency Virus SIVsmE660 Infection by DNA and Protein Coimmunization Regimens Combined with Different Toll-Like-Receptor-4-Based Adjuvants in Macaques — Supplemental material 

# Control of Heterologous Simian Immunodeficiency Virus SIVsmE660 Infection by DNA and Protein Coimmunization Regimens Combined with Different Toll-Like-Receptor-4-Based Adjuvants in Macaques

## Supplemental material

- Supplemental file 1 -

  Fig. S1 (Phenotypic analysis of PBMCs.)

  Fig. S2 (Induction of V2 antibody responses.)

  Fig. S3 (Linear peptide response analysis of plasma bAb.)

  Fig. S4 (Durability of vaccine-induced immune responses.)

  Fig. S5 (SIVsmE660 acquisition in TRIM-5α rhesus macaques.)

  Fig. S6 (Genetic analysis of T/F Env sequences.)

  Fig. S7 (Virus load measurements of SIVsmE660-infected animals.)

  Fig. S8 (Anamnestic SIV-specific T cell responses and association of cytotoxic SIV-specific effector memory T cells with control of viremia.)

  Table S1 (Macaque information.)

  Table S2 (Parameters measured by serum serology.)

  Table S3 (Association of humoral responses with virus acquisition and control of viremia.)

  Table S4 (Association of cellular immune responses and control of viremia.)

  PDF, 2.2M
