# Supplementary material for: Control of Heterologous Simian Immunodeficiency Virus SIVsmE660 Infection by DNA and Protein Coimmunization Regimens Combined with Different Toll-Like-Receptor-4-Based Adjuvants in Macaques
Source: J Virol. 2018 Jul 17;92(15):e00281-18. doi: 10.1128/JVI.00281-18 (PMC6052320; doi:10.1128/JVI.00281-18)
Supplement: Supplemental material [file JVI.00281-18_zjv015183736s1.pdf]

Supplementary Figure legends

Figure S1. Phenotypic analysis of PBMC. Plots show expression of the indicated markers on $CD4^+$ and $CD8^+$ T cells analyzed by flow cytometry at baseline (pre-vaccination), at 2 weeks after 2nd vaccination and at 10 weeks after the 3rd vaccination. Naïve controls (grey diamonds) and sham controls injected at month 6 (day of EP3) with TLR4+7 (black diamond) or TLR7+QS21 (open diamond) adjuvants are shown. Bars indicate median values.

Figure S2. Induction of V2 antibody responses. (A) Alignment of the amino acid (AA) sequence of the V1V2 region of SIV_{mac239} and $SIV_{smE660\text{-}GC7V}$, the V2 cyclic peptides (SIV_{smE543}) and SIV_{mac251} linear peptides 26-28 is shown. The numbering follows the SIVmac239 sequence. (B) Binding to each Env regions (detailed in Fig. S3) was measured as area-under-the-curve (AUC) and was calculated as percentage of the total AUC of Env gp120 (linear peptides 1-84). Adjusted p value are from 2-way ANOVA Holm-Sidak's multiple comparisons test. (C-E) bAb were measured to (C) cyclic SIV_{mac251} V2 by SPR assay and BIAcore; (D, E) SIV-BAMA to SIV_{mac251} gp70-V1V2 in (D) plasma and in (E) rectal and vaginal secretions. All plasma samples analyzed are from 2 weeks after the 3rd vaccination. Bars indicate median values.

Figure S3. Linear peptide response analysis of plasma bAb. The plasma samples of the individual animals were tested for their ability to bind to linear peptides spanning SIV_{mac251} gp120 (115 peptides, 20-mer overlapping by 14 AA) by standard ELISA. OD_{450} is reported after subtracting the mean OD_{450} of the values obtained from naive plasma samples.

Figure S4. Durability of vaccine-induced immune responses. Durability of SIV_{smE660} obtained at 2 and 18 weeks after the 3rd vaccination are shown for (A) plasma bAb; (B) NAb to SIVsmE660/2A5-VTRN; and (C) SIV-specific (Gag and Env M766) IFN-γ^+ T cell responses.

Figure S5. SIV_{smE660} acquisition in TRIM-5α R macaques. Kaplan–Meier curves of the number of SIV_{smE660} exposures to infection of vaccinees and controls carrying the TRIM-5α R^+ genotype with the TLR4+7 group (N=5, top panel) and the TLR4+QS21 group (N=5, lower panel) and the control animals (N=5). P value is from Gehan-Breslow-Wilcoxon test.

Figure S6. Genetic analysis of T/F Env sequences. Fifty-four informative sites were plotted as proportion of each AA compared to the consensus sequence. Residues 45 (T) and 47 (R), associated with neutralization resistance (54), showed the most prominent changes in the vaccinees compared to controls.

Figure S7. Virus load measurements of the SIV_{smE660}-infected animals. Virus load measurements (SIV RNA copies per ml in log) of the plasma are shown over the 25 weeks of follow-up. The animals of vaccine groups and controls are shown (N=12). Geometric mean of virus loads for animals with TRIM-5α R (N=5) and TRIM-5α M/S (N=7) genotype are shown for TLR4+7, TLR4+QS21 and controls as dotted (TRIM R) and solid (TRIM M/S) lines. t, marks macaque T073 (control group) that developed AIDS-like symptoms and died at week 18 post-infection before study end.

Figure S8. Anamnestic SIV-specific T cell responses and association of cytotoxic SIV-specific effector memory T cells with control of viremia. (A) Gag and (B) Env $CD4^+$ (open bar) and $CD8^+$ (black bar) T cell responses were measured at 2 weeks before challenge and 8 weeks post-infection. (C) Comparison of total SIV-specific (Gag and Env SIV_{smE660}-GC7V) IFN-γ^+ $GrzB^+$ effector memory T cells measured 2 weeks before challenge and at peak after infection (week 4 or week 8). P values are from Wilcoxon matched-pairs signed rank test. (D) Inverse correlations of total SIV-specific (Gag and SIV_{smE660}-GC7V) IFN-γ^+ $GrzB^+$ effector memory T cell measured 2 weeks after 3rd vaccination and VL at peak (left panel) and during early post-peak phase (weeks

2-4 post-infection). Spearman r and P values are shown.

TABLE S1. Macaque information

Group with adjuvant	Animal	Sex	Date of birth	TRIM-5α genotype	TRIM-5α[a]
TLR4+7	T065	Male	5/5/12	TFP/TFP	R
TLR4+7	T066	Male	4/16/12	Q/Q	S
TLR4+7	T069	Male	6/5/12	TFP/Q	M
TLR4+7	T071	Male	5/1/12	TFP/Q	M
TLR4+7	T074	Male	7/1/12	Q/Q	S
TLR4+7	T075	Male	3/30/12	TFP/CYPA	R
TLR4+7	T078	Male	3/30/12	Q/CYPA	M
TLR4+7	T092	Female	4/9/12	TFP/TFP	R
TLR4+7	T093	Female	5/27/12	TFP/CYPA	R
TLR4+7	T094	Female	4/16/12	TFP/Q	M
TLR4+7	T098	Female	4/20/12	TFP/TFP	R
TLR4+7	T099	Female	1/1/12	TFP/Q	M
TLR4+QS21	T072	Male	4/22/12	TFP/Q	M
TLR4+QS21	T076	Male	6/15/12	TFP/TFP	R
TLR4+QS21	T077	Male	4/19/12	Q/Q	S
TLR4+QS21	T079	Male	1/1/12	Q/Q	S
TLR4+QS21	T081	Male	4/27/12	TFP/CYPA	R
TLR4+QS21	T083	Male	9/1/12	TFP/Q	M
TLR4+QS21	T084	Male	3/16/12	Q/CYPA	M
TLR4+QS21	T085	Female	1/1/12	TFP/Q	M
TLR4+QS21	T086	Female	1/1/12	TFP/Q	M
TLR4+QS21	T088	Female	5/1/12	TFP/TFP	R
TLR4+QS21	T090	Female	7/17/12	TFP/CYPA	R
TLR4+QS21	T091	Female	7/1/12	TFP/TFP	R
Control/ TLR4+7	T080	Male	1/1/12	TFP/CYPA	R
Control/ TLR4+7	T082	Male	6/6/12	Q/Q	S
Control/ TLR4+7	T095	Female	4/10/12	TFP/TFP	R
Control/ TLR4+QS21	T073	Male	2/28/12	TFP/Q	M
Control/ TLR4+QS21	T087	Female	1/1/12	Q/CYPA	M
Control/ TLR4+QS21	T096	Female	6/6/12	TFP/Q	M
Control naive	T067	Male	4/6/12	TFP/Q	M
Control naive	T068	Male	4/5/12	TFP/TFP	R
Control naive	T070	Male	4/19/12	TFP/TFP	R
Control naive	T089	Female	1/1/12	Q/CYPA	M
Control naive	T097	Female	4/5/12	Q/Q	S
Control naive	T100	Female	5/10/12	TFP/CYPA	R

[a]R, TRIM-5α most resistant; M, TRIM-5α moderate resistant; S, TRIM-5α, most susceptible. Animals with M and S genotypes are combined in this study and referred to as M/S.

Table S2. Parameters measured by serum serology

Feature	Feature Name	Feature Description
**NK activity**	MIP-1β	NK cell MIP-1β activation
	IFN-γ	NK cell IFN-γ activation
	CD107a	NK cell CD107a activation
**Effector Function**	ADCD	antibody-dependent complement deposition
	ADCC	antibody-dependent cell-mediated cytotoxicity
	ADCP	antibody-dependent cellular phagocytosis
	ADNP	antibody-dependent neutrophil phagocytosis
**Individual Glycan Structures:**		
M766-specific IgG	G0F	agalactosylated (G0), fucosylated (F)
	G1B	galactosylated (G1), bisecting (B)
	G1S1	galactosylated (G1), sialylated (S1)
	G1S1F	galactosylated (G1), sialylated (S1), fucosylated (F)
	G2	di-galactosylated (G2)
	G2F	di-galactosylated (G2), fucosylated (F)
	G2FB	di-galactosylated (G2), fucosylated (F), bisecting (B)
	G2S1	di-galactosylated (G2), sialylated (S1), fucosylated (F)
	G2S1FB	di-galactosylated (G2), sialylated (S1), fucosylated (F), bisecting (B)
	G2S2	di-galactosylated (G2), di-sialylated (S2)
	G2S2B	di-galactosylated (G2), di-sialylated (S2), bisecting (B)
	G2S2F	di-galactosylated (G2), di-sialylated (S2), fucosylated (F)
	G2S2FB	di-galactosylated (G2), di-sialylated (S2), fucosylated (F), bisecting (B)

TABLE S3. Association of humoral responses with virus acquisition and control of viremia

Challenge phase	Antibody determination[a]	Animals	Spearman r	p (two-tailed)	p	Graph shown in Figure
**acquisition**	gp140smE660[b]	TLR4+7	0.6735	0.0205	*	4D
	NAbmac251.6[b]	Vaccinees	0.4233	0.0393	*	
	NAbmac251.6[b]	TLR4+7	0.6020	0.0429	*	
	NAbsmE660/BR-CG7G.IR[b]	Vaccinees	0.6931	0.0002	***	4E
	NAbsmE660/BR-CG7G.IR1[b]	TLR4+7	0.7149	0.0127	*	
	NAbsmE660/BR-CG7G.IR1[b]	TLR4+7	0.5667	0.0586		
	NAbsmE660/2A5-VTRN[b]	Vaccinees	0.5728	0.0034	**	4F
	NAbsmE660/2A5-VTRN[b]	TLR4+7	0.8541	0.0010	**	
	NAbsmE660/2A5-VTRN[b]	TLR4+7	0.6547	0.0251	*	
**peak**	gp130 mac251[c]	TRIM-5α M/S	-0.7904	0.0038	**	
	V1V2 gp70-mac251[b]	TRIM-5α M/S	-0.5385	0.0500	*	5C
	V1V2 gp70-mac251[c]	TRIM-5α M/S	-0.7113	0.0124	*	5D
	V1V2 gp70-mac239[c]	TRIM-5α M/S	-0.6409	0.0290	*	
**AUC 2-4**	gp130 mac251[c]	TRIM-5α M/S	-0.6744	0.0205	*	
	cyclic V2 E543 (F)[b]	TRIM-5α M/S	-0.6123	0.0224	*	
	cyclic V2 E543 (S)[b]	TRIM-5α M/S	-0.6703	0.0107	*	5E
	cyclic V2 mac251 (F)[b]	TRIM-5α M/S	-0.6659	0.0113	*	
	cyclic V2 mac251 (S)[b]	TRIM-5α M/S	-0.6967	0.0072	**	
	V1V2 gp70-mac251[b]	TRIM-5α M/S	-0.5429	0.0479	*	
	V1V2 gp70-mac251[c]	TRIM-5α M/S	-0.6479	0.0268	*	
**AUC 6-25**	gp130 mac251[c]	TRIM-5α M/S	-0.7686	0.0055	**	
	V1V2 gp70-mac239[c]	TRIM-5α M/S	-0.6549	0.0248	*	
	V1V2 gp70-mac251[c]	TRIM-5α M/S	-0.7465	0.0074	**	
	cyclic V2 E543(F)[b]	TRIM-5α M/S	-0.5736	0.0349	*	
	cyclic V2 E543(S)[b]	TRIM-5α M/S	-0.6527	0.0136	*	5F
	cyclic V2 mac251(F)[b]	TRIM-5α M/S	-0.6215	0.0201	*	
	cyclic V2 mac251(S)[b]	TRIM-5α M/S	-0.662	0.0121	*	

[a]Antibody determination at 2 weeks after 3rd vaccination.
[b]Antibody determination in plasma.
[c]Antibody determination in rectal mucosal samples.

TABLE S4. Association of cellular immune responses and control of viremia

Challenge phases	Antigen	Cellular Immunity[a, b]	Spearman r	P (two-tailed)	P	Graph shown in Figure
**Peak**	Gag+Env	$CD3^+$	-0.6088	0.0236	*	
	Gag	$CD3^+$	-0.6923	0.0077	**	6C
	Env	$CD3^+$	-0.5692	0.0366	*	6D
**AUC wk 2-4**	Gag+Env	$CD3^+$	-0.5824	0.0318	*	
	Gag	$CD3^+$	-0.7363	0.0037	**	
	Gag	$CD3^+CD4^+$	-0.6271	0.0186	*	
	Env	$CD3^+$ $CD95^+$ $CD28^+$ $GrzB^+$	-0.5585	0.0411	*	
**AUC wk 6-25**	Gag	$CD3^+$	-0.5516	0.0438	*	6E

[a]Vaccinees with the TRIM-5α M/S genotype.
[b]Cellular responses measured 2 weeks before 1st virus exposure.

Figure S1

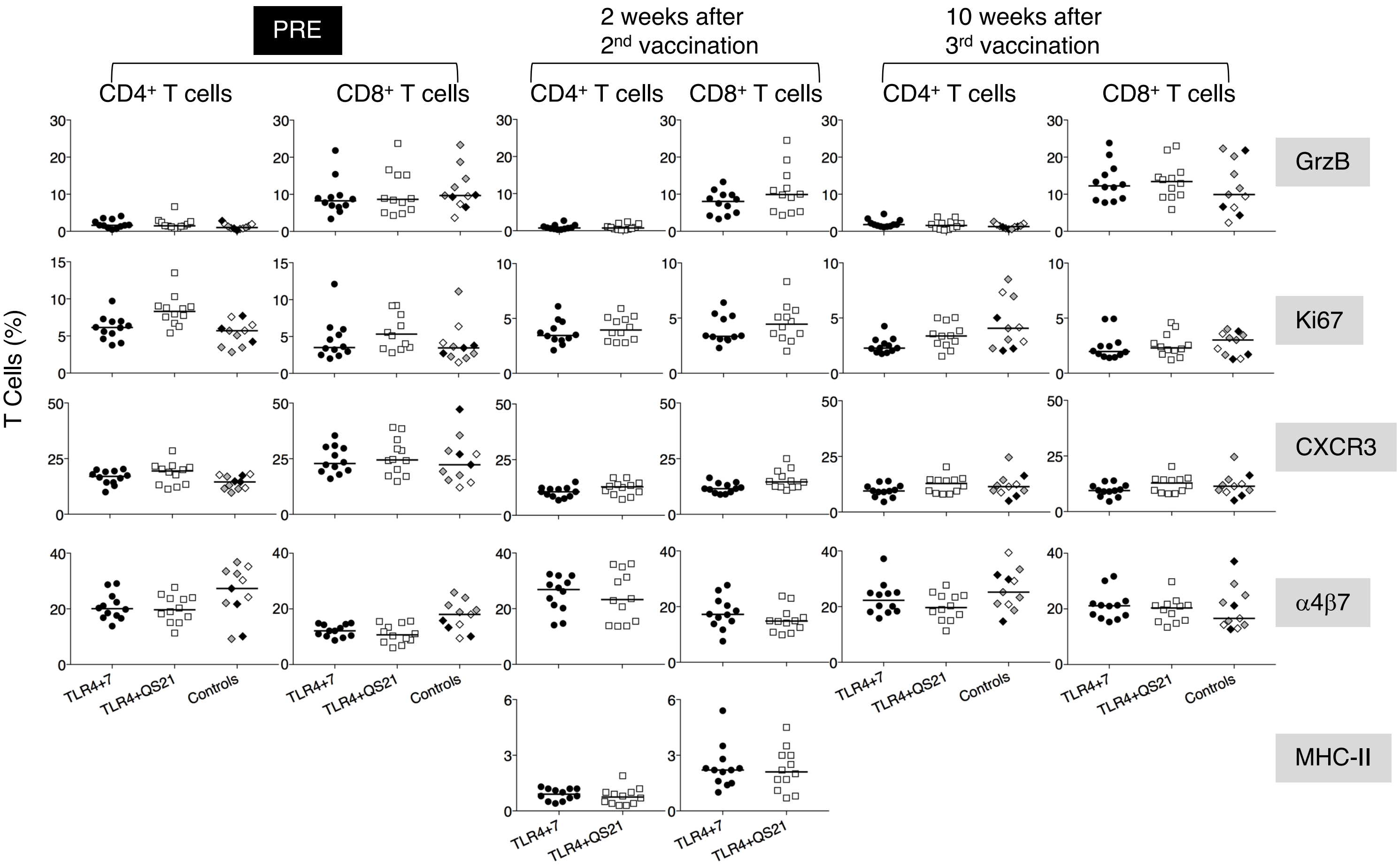

PRE
2 weeks after 2nd vaccination
10 weeks after 3rd vaccination
CD4+ T cells
CD8+ T cells
T Cells (%)
GrzB
Ki67
CXCR3
α4β7
MHC-II
TLR4+7
TLR4+QS21
Controls

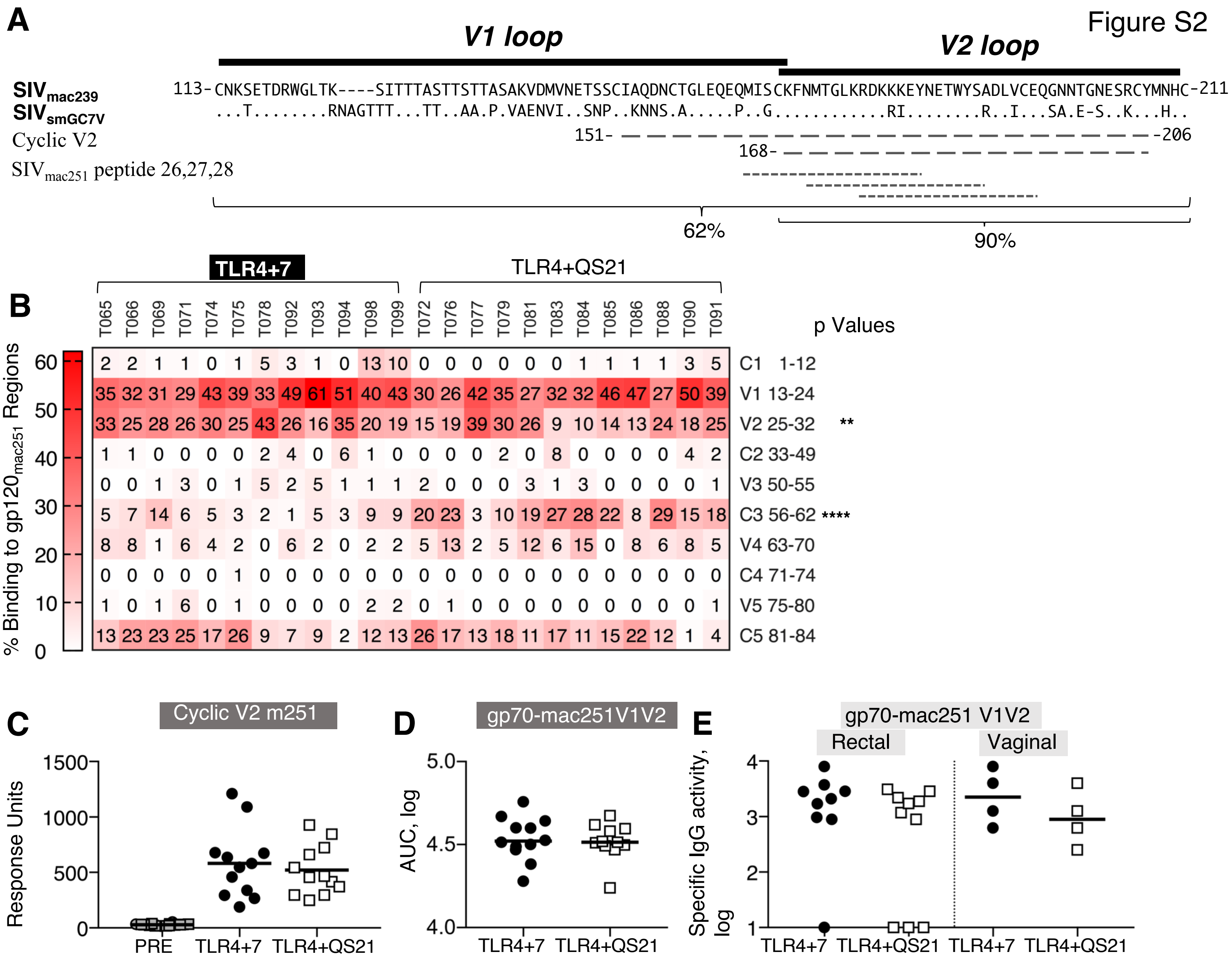
A
V1 loop
V2 loop
SIVmac239
SIVsmGC7V
113-CNKSETDRWGLTK----SITTTASTTSTTASAKVDMVNETSSCIAQDNCTGLEQEQMISCKFNMTGLKRDKKKEYNETWYSADLVCEQGNNTGNESRCYMNHC-211
...T........RNAGTTT...TT..AA.P.VAENVI..SNP..KNNS.A.....P..G...........RI........R..I...SA.E-S..K...H..
Cyclic V2
151-
-206
168-
SIVmac251 peptide 26,27,28
62%
90%
B
TLR4+7
TLR4+QS21
T065 T066 T069 T071 T074 T075 T078 T092 T093 T094 T098 T099 T072 T076 T077 T079 T081 T083 T084 T085 T086 T088 T090 T091
p Values
% Binding to gp120mac251 Regions
60 50 40 30 20 10 0
2 2 1 1 0 1 5 3 1 0 13 10 0 0 0 0 0 0 1 1 1 1 3 5 C1 1-12
35 32 31 29 43 39 33 49 61 51 40 43 30 26 42 35 27 32 32 46 47 27 50 39 V1 13-24
33 25 28 26 30 25 43 26 16 35 20 19 15 19 39 30 26 9 10 14 13 24 18 25 V2 25-32 **
1 1 0 0 0 0 2 4 0 6 1 0 0 0 0 2 0 8 0 0 0 0 4 2 C2 33-49
0 0 1 3 0 1 5 2 5 1 1 1 2 0 0 0 3 1 3 0 0 0 0 1 V3 50-55
5 7 14 6 5 3 2 1 5 3 9 9 20 23 3 10 19 27 28 22 8 29 15 18 C3 56-62 ****
8 8 1 6 4 2 0 6 2 0 2 2 5 13 2 5 12 6 15 0 8 6 8 5 V4 63-70
0 0 0 0 0 1 0 0 0 0 0 0 0 0 0 0 0 0 0 0 0 0 0 0 C4 71-74
1 0 1 6 0 1 0 0 0 0 2 2 0 1 0 0 0 0 0 0 0 0 0 1 V5 75-80
13 23 23 25 17 26 9 7 9 2 12 13 26 17 13 18 11 17 11 15 22 12 1 4 C5 81-84
C
Cyclic V2 m251
Response Units
1500 1000 500 0
PRE TLR4+7 TLR4+QS21
D
gp70-mac251V1V2
AUC, log
5.0 4.5 4.0
TLR4+7 TLR4+QS21
E
gp70-mac251 V1V2
Rectal
Vaginal
Specific IgG activity, log
4 3 2 1
TLR4+7 TLR4+QS21 TLR4+7 TLR4+QS21
Figure S2

Figure S3

Peptide	T065	T066	T069	T071	T074	T075	T078	T092	T093	T094	T098	T099	T072	T076	T077	T079	T081	T083	T084	T085	T086	T088	T090	T091	Region
	TLR4+7												TLR4+QS21												
1	0.00	0.00	0.00	0.00	0.00	0.00	0.00	0.00	0.00	0.00	0.01	0.00	0.00	0.00	0.00	0.00	0.00	0.00	0.00	0.00	0.00	0.00	0.00	0.02	C1 1-12
2	0.00	0.00	0.00	0.00	0.00	0.00	0.00	0.00	0.00	0.00	0.00	0.00	0.00	0.00	0.00	0.00	0.00	0.00	0.00	0.00	0.00	0.00	0.00	0.00	
3	0.00	0.24	0.00	0.00	0.00	0.00	0.00	0.00	0.00	0.00	0.00	0.00	0.00	0.04	0.00	0.00	0.00	0.00	0.00	0.00	0.00	0.00	0.03	0.01	
4	0.00	0.13	0.04	0.00	0.00	0.00	0.05	0.03	0.00	0.02	0.00	0.10	0.00	0.05	0.00	0.00	0.02	0.01	0.03	0.00	0.08	0.00	0.05	0.08	
5	0.00	0.00	0.00	0.00	0.00	0.00	0.00	0.00	0.00	0.00	0.00	0.00	0.00	0.00	0.00	0.00	0.00	0.00	0.01	0.02	0.02	0.00	0.00	0.02	
6	0.10	0.09	0.00	0.00	0.00	0.00	0.00	0.02	0.02	0.03	0.04	0.04	0.02	0.01	0.00	0.00	0.00	0.02	0.03	0.02	0.06	0.17	0.00	0.06	
7	0.00	0.00	0.00	0.00	0.00	0.00	0.00	0.00	0.00	0.00	0.18	0.11	0.00	0.00	0.00	0.00	0.07	0.00	0.00	0.02	0.00	0.02	0.00	0.63	
8	0.00	0.00	0.00	0.00	0.00	0.00	0.00	0.01	0.00	0.00	0.02	0.01	0.00	0.01	0.01	0.00	0.00	0.00	0.00	0.01	0.00	0.00	0.00	0.01	
9	0.00	0.00	0.03	0.02	0.00	0.00	0.04	0.10	0.07	0.02	1.25	0.90	0.00	0.03	0.00	0.00	0.00	0.00	0.00	0.03	0.08	0.02	0.00	0.10	
10	0.00	0.00	0.03	0.00	0.00	0.00	0.00	0.00	0.00	0.00	0.65	0.40	0.00	0.00	0.00	0.02	0.00	0.00	0.00	0.02	0.03	0.01	0.04	0.03	
11	0.14	0.00	0.10	0.04	0.00	0.16	0.12	0.02	0.00	0.00	0.63	0.36	0.04	0.02	0.00	0.03	0.03	0.02	0.12	0.03	0.00	0.00	0.30	0.08	
12	0.28	0.02	0.03	0.01	0.00	0.17	0.11	0.04	0.00	0.09	0.01	0.00	0.00	0.00	0.00	0.00	0.00	0.00	0.05	0.02	0.00	0.00	0.00	0.02	
13	0.00	0.02	0.01	0.02	0.00	0.02	0.01	0.01	0.00	0.03	0.02	0.01	0.00	0.01	0.00	0.00	0.00	0.01	0.00	0.00	0.00	0.00	0.00	0.02	V1 13-24
14	0.04	0.00	0.01	0.03	0.00	0.04	0.00	0.01	0.01	0.07	0.03	0.03	0.00	0.04	0.00	0.00	0.00	0.02	0.00	0.00	0.00	0.01	0.00	0.02	
15	0.00	0.00	0.02	0.00	0.00	0.03	0.01	0.00	0.07	0.12	0.26	0.19	0.00	0.03	0.00	0.00	0.01	0.17	0.02	0.00	1.02	0.03	0.01	0.27	
16	2.48	2.76	2.59	0.57	1.45	3.95	0.50	1.02	2.16	2.66	2.93	2.98	1.75	3.86	2.15	3.29	3.93	2.21	2.41	2.92	3.45	2.60	3.03	3.75	
17	2.42	3.95	3.76	1.18	2.87	3.95	1.08	2.05	2.50	3.43	3.16	3.06	2.30	3.71	2.03	3.65	3.92	2.98	2.85	3.31	3.92	2.46	3.15	3.92	
18	0.19	0.22	0.13	0.06	0.12	0.23	0.04	0.08	0.06	0.12	0.20	0.18	0.00	0.08	0.02	0.19	0.22	0.12	0.13	0.07	0.29	0.05	0.03	0.19	
19	0.00	0.01	0.00	0.00	0.01	0.00	0.01	0.40	0.00	0.01	0.01	0.01	0.00	0.00	0.00	0.00	0.00	0.00	0.00	0.00	0.01	0.00	0.00	0.03	
20	0.03	0.01	0.04	0.00	0.00	0.01	0.02	0.03	0.00	0.01	0.04	0.00	0.00	0.00	0.00	0.00	0.00	0.03	0.00	0.00	0.00	0.00	0.00	0.08	
21	0.02	0.01	0.06	0.00	0.00	0.01	0.02	0.06	0.00	0.03	0.68	0.62	0.00	0.02	0.00	0.00	0.00	0.00	0.00	0.00	1.36	0.00	0.00	0.02	
22	0.18	0.01	0.06	0.00	0.00	0.03	0.01	0.05	0.08	0.11	1.11	0.97	0.06	0.03	0.00	0.00	0.02	0.00	0.00	0.00	0.80	0.02	0.10	0.02	
23	0.00	0.00	0.01	0.00	0.00	0.01	0.03	0.02	0.00	0.13	0.04	0.02	0.00	0.37	0.00	0.00	0.00	0.17	0.00	0.00	0.01	0.00	0.02	0.00	
24	0.14	0.02	0.04	0.58	0.00	0.02	0.01	0.02	0.00	0.06	0.21	0.16	0.00	0.28	0.00	0.00	0.00	0.10	0.00	0.02	0.04	0.00	0.11	0.30	
25	1.07	0.12	0.34	0.48	0.02	0.70	0.23	0.57	0.00	1.15	0.62	0.64	0.94	2.67	0.02	0.02	0.00	0.26	0.06	0.08	0.14	0.11	0.27	0.84	V2 25-32
26	1.14	1.49	1.83	0.15	1.81	2.12	0.21	1.11	0.73	2.06	0.64	0.51	1.02	3.49	2.72	1.62	3.76	0.81	0.48	0.74	2.23	2.97	0.93	2.41	
27	2.09	2.53	2.45	1.03	0.48	1.02	1.26	0.30	0.39	0.70	1.88	1.65	0.22	0.80	1.03	2.26	2.57	0.37	0.66	0.56	0.52	1.21	0.90	1.59	
28	1.33	1.29	1.43	0.45	0.76	1.75	0.65	0.22	0.11	1.13	1.12	0.95	0.32	0.32	0.11	2.08	1.56	0.18	0.42	0.54	0.07	0.41	0.38	0.70	
29	0.00	0.04	0.12	0.03	0.00	0.03	0.02	0.05	0.00	0.07	0.08	0.06	0.00	0.04	0.00	0.05	0.03	0.04	0.00	0.03	0.00	0.04	0.00	0.09	
30	0.00	0.00	0.08	0.00	0.00	0.01	0.00	0.03	0.01	0.02	0.04	0.04	0.00	0.00	0.00	0.00	0.00	0.02	0.00	0.00	0.00	0.00	0.00	0.04	
31	0.00	0.06	0.06	0.02	0.08	0.00	0.02	0.03	0.02	0.03	0.07	0.06	0.00	0.05	0.01	0.00	0.00	0.02	0.02	0.05	0.00	0.00	0.00	0.08	
32	0.00	0.03	0.01	0.00	0.00	0.00	0.00	0.00	0.00	0.02	0.03	0.00	0.00	0.00	0.00	0.00	0.00	0.00	0.04	0.00	0.00	0.00	0.00	0.00	
33	0.10	0.04	0.02	0.00	0.00	0.00	0.00	0.07	0.00	0.00	0.01	0.00	0.00	0.00	0.00	0.00	0.00	0.00	0.00	0.04	0.00	0.00	0.02	0.00	C2 33-49
34	0.03	0.02	0.02	0.00	0.00	0.00	0.04	0.02	0.00	0.00	0.02	0.01	0.00	0.04	0.00	0.00	0.05	0.01	0.01	0.00	0.00	0.00	0.00	0.02	
35	0.04	0.01	0.00	0.00	0.02	0.00	0.01	0.01	0.00	0.00	0.01	0.00	0.00	0.01	0.00	0.00	0.00	0.00	0.03	0.00	0.00	0.00	0.00	0.04	
36	0.00	0.00	0.00	0.00	0.00	0.00	0.00	0.00	0.00	0.00	0.00	0.00	0.00	0.00	0.00	0.00	0.00	0.00	0.00	0.00	0.00	0.00	0.00	0.12	
37	0.00	0.00	0.00	0.00	0.00	0.00	0.00	0.00	0.00	0.00	0.00	0.00	0.00	0.00	0.00	0.00	0.00	0.00	0.00	0.00	0.00	0.00	0.04	0.06	
38	0.00	0.04	0.00	0.00	0.00	0.00	0.00	0.08	0.00	0.00	0.04	0.04	0.00	0.03	0.00	0.13	0.00	0.18	0.00	0.00	0.03	0.00	0.09	0.06	
39	0.00	0.02	0.02	0.00	0.01	0.00	0.00	0.19	0.00	0.73	0.04	0.05	0.00	0.05	0.00	0.18	0.00	0.34	0.00	0.06	0.07	0.04	0.27	0.07	
40	0.00	0.02	0.00	0.00	0.00	0.00	0.00	0.00	0.01	0.05	0.04	0.03	0.00	0.02	0.00	0.00	0.00	0.79	0.00	0.01	0.07	0.00	0.08	0.04	
41	0.00	0.00	0.00	0.00	0.00	0.00	0.00	0.00	0.00	0.00	0.00	0.00	0.00	0.00	0.00	0.00	0.00	0.00	0.00	0.00	0.00	0.00	0.00	0.01	
42	0.00	0.02	0.00	0.00	0.00	0.00	0.00	0.00	0.00	0.00	0.00	0.00	0.00	0.00	0.00	0.00	0.00	0.00	0.00	0.00	0.00	0.00	0.00	0.00	
43	0.00	0.00	0.00	0.00	0.00	0.00	0.00	0.00	0.00	0.00	0.00	0.00	0.00	0.00	0.00	0.00	0.00	0.01	0.00	0.00	0.00	0.00	0.00	0.01	
44	0.00	0.05	0.00	0.00	0.00	0.00	0.00	0.00	0.00	0.00	0.00	0.00	0.00	0.00	0.00	0.00	0.00	0.00	0.00	0.00	0.00	0.00	0.00	0.05	
45	0.02	0.00	0.00	0.00	0.00	0.00	0.00	0.00	0.00	0.00	0.00	0.00	0.00	0.00	0.00	0.00	0.08	0.01	0.00	0.00	0.00	0.00	0.00	0.01	
46	0.02	0.02	0.00	0.00	0.02	0.00	0.02	0.00	0.00	0.01	0.00	0.00	0.00	0.00	0.00	0.00	0.00	0.00	0.00	0.00	0.00	0.00	0.00	0.01	
47	0.00	0.01	0.01	0.00	0.00	0.00	0.02	0.00	0.00	0.01	0.02	0.01	0.00	0.06	0.00	0.00	0.00	0.01	0.03	0.00	0.00	0.00	0.01	0.01	
48	0.04	0.02	0.01	0.00	0.00	0.00	0.00	0.00	0.02	0.00	0.00	0.00	0.00	0.02	0.00	0.00	0.00	0.00	0.00	0.00	0.00	0.00	0.00	0.00	
49	0.02	0.02	0.11	0.01	0.01	0.05	0.01	0.02	0.00	0.02	0.02	0.01	0.00	0.02	0.02	0.00	0.00	0.00	0.00	0.00	0.00	0.00	0.00	0.00	
50	0.00	0.03	0.10	0.00	0.07	0.02	0.02	0.26	0.72	0.00	0.13	0.16	0.00	0.00	0.04	0.00	1.18	0.13	0.64	0.00	0.00	0.00	0.00	0.00	V3 50-55
51	0.03	0.04	0.10	0.22	0.04	0.30	0.23	0.02	0.03	0.19	0.20	0.15	0.29	0.08	0.00	0.00	0.23	0.00	0.12	0.00	0.16	0.15	0.00	0.09	
52	0.00	0.02	0.03	0.00	0.00	0.00	0.03	0.00	0.00	0.00	0.00	0.00	0.00	0.00	0.00	0.00	0.00	0.07	0.00	0.00	0.00	0.00	0.00	0.03	
53	0.00	0.00	0.00	0.00	0.00	0.00	0.00	0.04	0.00	0.00	0.00	0.00	0.00	0.00	0.00	0.00	0.00	0.00	0.00	0.00	0.00	0.00	0.03	0.05	
54	0.00	0.01	0.00	0.00	0.00	0.00	0.00	0.00	0.00	0.00	0.00	0.00	0.00	0.00	0.00	0.00	0.00	0.02	0.00	0.00	0.00	0.00	0.00	0.05	
55	0.00	0.03	0.00	0.00	0.00	0.00	0.01	0.00	0.00	0.02	0.00	0.00	0.00	0.00	0.00	0.00	0.00	0.00	0.00	0.00	0.00	0.00	0.00	0.03	
56	0.00	0.02	0.00	0.00	0.00	0.00	0.00	0.00	0.00	0.00	0.00	0.00	0.02	0.01	0.00	0.00	0.00	0.00	0.00	0.00	0.01	0.00	0.00	0.19	C3 56-62
57	0.32	0.64	1.75	0.10	0.21	0.31	0.04	0.03	0.03	0.18	0.80	0.64	0.78	1.94	0.08	0.79	2.09	2.04	1.30	1.31	0.94	2.28	0.69	1.30	
58	0.39	0.62	0.87	0.15	0.23	0.31	0.04	0.06	0.04	0.16	0.79	0.73	0.95	3.22	0.15	1.02	1.96	1.70	1.61	1.11	0.53	2.33	0.80	1.89	
59	0.11	0.34	0.46	0.16	0.06	0.08	0.03	0.03	0.31	0.06	0.30	0.31	1.00	2.03	0.09	0.17	1.79	1.02	1.82	0.64	0.26	0.97	0.45	0.51	
60	0.00	0.00	0.02	0.00	0.00	0.00	0.00	0.00	0.00	0.00	0.00	0.00	0.00	0.03	0.00	0.00	0.00	0.01	0.02	0.00	0.00	0.00	0.00	0.06	
61	0.00	0.00	0.00	0.00	0.00	0.00	0.00	0.00	0.00	0.00	0.00	0.00	0.00	0.00	0.00	0.00	0.00	0.00	0.00	0.00	0.00	0.00	0.00	0.03	
62	0.00	0.00	0.00	0.00	0.04	0.00	0.00	0.01	0.00	0.00	0.00	0.00	0.00	0.00	0.00	0.00	0.00	0.00	0.00	0.00	0.00	0.00	0.00	0.02	
63	0.00	0.00	0.00	0.00	0.00	0.00	0.00	0.00	0.02	0.00	0.01	0.00	0.00	0.00	0.00	0.00	0.00	0.00	0.00	0.00	0.00	0.00	0.00	0.01	V4 63-70
64	0.00	0.04	0.02	0.00	0.00	0.00	0.00	0.00	0.00	0.00	0.00	0.00	0.01	0.00	0.00	0.00	0.00	0.00	0.00	0.00	0.00	0.00	0.00	0.05	
65	0.00	0.02	0.01	0.00	0.00	0.00	0.02	0.02	0.00	0.00	0.00	0.00	0.00	0.00	0.00	0.00	0.00	0.02	0.00	0.00	0.00	0.00	0.00	0.00	
66	0.06	0.23	0.00	0.00	0.03	0.00	0.00	0.01	0.03	0.00	0.00	0.00	0.00	0.73	0.04	0.00	0.00	0.05	0.00	0.00	0.00	0.12	0.00	0.00	
67	0.46	0.94	0.01	0.34	0.27	0.00	0.00	0.15	0.05	0.00	0.13	0.13	0.45	2.83	0.17	0.66	2.17	0.85	1.68	0.08	0.89	0.83	0.77	0.43	
68	0.68	0.47	0.18	0.13	0.09	0.46	0.02	0.25	0.07	0.04	0.24	0.26	0.20	0.44	0.00	0.29	1.38	0.11	0.71	0.02	0.62	0.20	0.29	0.36	
69	0.00	0.01	0.00	0.00	0.00	0.00	0.00	0.01	0.00	0.00	0.01	0.02	0.01	0.07	0.00	0.00	0.15	4.00e-003	0.07	0.00	0.21	0.00	0.00	0.13	
70	0.00	0.00	0.00	0.00	0.00	0.00	0.00	0.00	0.00	0.00	0.00	0.00	0.00	0.02	0.00	0.00	0.04	0.00	0.04	0.00	0.09	0.00	0.00	0.06	
71	0.01	0.01	0.01	0.00	0.00	0.00	0.00	0.00	0.01	0.00	0.01	0.00	0.05	0.13	0.00	0.00	0.00	0.02	0.01	0.00	0.03	0.07	0.00	0.04	C4 71-74
72	0.01	0.00	0.01	0.00	0.00	0.03	0.00	0.00	0.01	0.00	0.00	0.00	0.00	0.00	0.00	0.00	0.00	0.00	0.00	0.00	0.00	0.01	0.00	0.00	
73	0.04	0.00	0.01	0.00	0.02	0.28	0.00	0.03	0.00	0.00	0.00	0.00	0.00	0.00	0.00	0.15	0.00	0.00	0.00	0.00	0.08	0.00	0.00	0.00	
74	0.00	0.03	0.01	0.00	0.00	0.00	0.02	0.02	0.00	0.04	0.00	0.00	0.00	0.00	0.00	0.00	0.00	0.00	0.00	0.00	0.00	0.00	0.04	0.00	
75	0.00	0.04	0.00	0.00	0.00	0.00	0.00	0.00	0.00	0.00	0.00	0.00	0.00	0.00	0.00	0.00	0.00	0.00	0.00	0.00	0.00	0.00	0.00	0.00	V5 75-80
76	0.00	0.04	0.00	0.00	0.00	0.00	0.00	0.00	0.00	0.00	0.00	0.00	0.00	0.00	0.00	0.00	0.00	0.08	0.00	0.00	0.00	0.00	0.00	0.01	
77	0.00	0.00	0.00	0.00	0.00	0.01	0.00	0.00	0.00	0.00	0.00	0.00	0.00	0.00	0.00	0.00	0.00	0.01	0.00	0.00	0.00	0.00	0.00	0.02	
78	0.07	0.00	0.00	0.00	0.00	0.17	0.00	0.00	0.00	0.00	0.00	0.00	0.00	0.03	0.00	0.00	0.00	0.00	0.00	0.00	0.00	0.00	0.00	0.08	
79	0.03	0.00	0.00	0.00	0.00	0.02	0.00	0.01	0.00	0.00	0.02	0.00	0.00	0.03	0.00	0.00	0.01	0.01	0.02	0.00	0.00	0.00	0.00	0.07	
80	0.07	0.02	0.43	0.83	0.01	0.11	0.01	0.00	0.02	0.17	0.88	0.88	0.02	0.54	0.00	0.19	0.00	0.10	0.08	0.02	0.02	0.00	0.00	0.09	
81	0.44	0.08	2.67	1.16	0.12	0.00	0.01	0.09	0.08	0.28	1.88	1.67	0.31	0.74	0.08	1.15	0.13	0.47	0.71	0.11	0.50	0.00	0.10	0.89	C5 81-84
82	0.13	1.47	2.49	0.98	0.18	0.93	0.02	0.07	0.29	0.04	1.01	1.03	0.73	0.98	0.12	1.31	0.52	0.82	0.41	0.29	1.72	0.46	0.02	0.41	
83	1.56	2.74	0.94	0.24	1.46	3.78	0.35	0.41	0.38	0.05	0.45	0.49	2.62	3.80	1.18	1.71	2.80	1.64	1.18	1.64	2.46	1.56	0.00	0.10	
84	0.26	1.25	0.50	0.06	0.07	1.72	0.16	0.00	0.08	0.02	0.17	0.17	0.10	0.34	0.00	0.05	0.00	0.84	0.00	0.24	1.38	0.56	0.00	0.02	
85	0.00	0.07	0.11	0.00	0.00	0.04	0.01	0.00	0.03	0.00	0.18	0.18	0.11	0.33	0.00	0.02	0.00	0.88	0.28	0.05	1.58	0.20	0.00	0.05	Fusion Peptide 85-88
86	0.03	0.05	0.04	0.00	0.00	0.00	0.00	0.00	0.04	0.00	0.08	0.08	0.02	0.04	0.00	0.00	0.00	0.63	0.00	0.00	0.59	0.10	0.00	0.00	
87	0.00	0.07	0.04	0.00	0.00	0.00	0.00	0.00	0.00	0.00	0.02	0.02	0.00	0.00	0.00	0.00	0.00	0.06	0.00	0.00	0.07	0.00	0.06	0.00	
88	0.00	0.06	0.05	0.00	0.00	0.08	0.00	0.00	0.00	0.00	0.11	0.10	0.02	0.09	0.00	0.00	0.00	0.18	0.00	0.00	0.15	0.02	0.00	0.02	
89	0.00	0.00	0.19	0.00	0.00	0.00	0.00	0.00	0.00	0.00	0.00	0.00	0.00	0.00	0.00	0.00	0.00	0.06	0.00	0.00	0.07	0.00	0.00	0.00	N-Heptad 89-95
90	0.00	0.06	0.88	0.00	0.00	0.02	0.00	0.01	0.00	0.00	0.27	0.27	0.00	0.01	0.00	0.00	0.00	0.11	0.00	0.00	0.31	0.00	0.02	0.00	
91	0.00	0.05	0.14	0.00	0.00	0.01	0.00	0.00	0.00	0.00	0.02	0.02	0.02	0.00	0.00	0.00	0.00	0.05	0.00	0.00	0.03	0.00	0.00	0.00	
92	0.00	0.09	0.11	0.00	0.01	0.01	0.00	0.00	0.00	0.00	0.02	0.02	0.00	0.00	0.00	0.00	0.00	0.01	0.00	0.00	0.03	0.00	0.00	0.00	
93	0.00	0.08	0.01	0.00	0.00	0.00	0.00	0.00	0.00	0.00	0.00	0.00	0.00	0.00	0.00	0.00	0.00	0.02	0.00	0.00	0.00	0.00	0.00	0.02	
94	0.00	0.01	0.08	0.00	0.00	0.00	0.00	0.00	0.00	0.00	0.00	0.00	0.00	0.09	0.00	0.00	0.00	0.00	0.00	0.00	0.00	0.00	0.00	0.03	
95	0.00	0.03	0.04	0.00	0.00	0.02	0.00	0.00	0.00	0.00	0.00	0.01	0.00	0.00	0.00	0.00	0.00	0.02	0.00	0.00	0.03	0.00	0.00	0.02	
96	0.00	0.04	0.04	0.00	0.00	0.00	0.01	0.00	0.00	0.03	0.01	0.01	0.00	0.01	0.00	0.00	0.00	0.21	0.00	0.10	0.03	0.20	0.00	0.12	gp41ID 96-102
97	0.00	0.28	0.02	0.04	0.03	0.01	0.01	0.01	0.01	0.02	0.00	0.00	0.00	0.00	0.00	0.00	0.00	0.00	0.35	0.25	0.00	0.02	0.00	0.11	
98	0.00	0.15	0.22	0.06	0.02	0.00	0.02	0.01	0.02	0.05	0.00	0.00	0.00	0.25	0.00	0.00	0.00	0.00	0.07	0.17	0.06	0.08	0.01	0.00	
99	0.00	0.01	0.00	0.00	0.03	0.00	0.03	0.02	0.04	0.00	0.00	0.00	0.00	0.00	0.00	0.00	0.00	0.00	0.00	0.00	0.00	0.00	0.00	0.00	
100	0.00	0.03	0.10	0.01	0.00	0.00	0.02	0.02	0.01	0.00	0.00	0.00	0.00	0.00	0.00	0.00	0.00	0.02	0.00	0.00	0.00	0.00	0.00	0.01	
101	0.01	0.03	0.02	0.00	0.00	0.00	0.00	0.01	0.02	0.00	0.00	0.00	0.00	0.00	0.00	0.00	0.00	0.01	0.00	0.03	0.00	0.00	0.00	0.00	
102	0.00	0.01	0.00	0.00	0.01	0.02	0.04	0.06	0.02	0.00	0.01	0.00	0.00	0.00	0.00	0.00	0.00	0.00	0.00	0.00	0.00	0.00	0.00	0.00	
103	0.00	0.00	0.00	0.02	0.00	0.02	0.01	0.06	0.05	0.00	0.00	0.00	0.00	0.00	0.00	0.00	0.00	0.00	0.01	0.00	0.00	0.00	0.00	0.00	C-Heptad 103-109
104	0.00	0.02	0.05	0.01	0.00	0.08	0.02	0.05	0.02	0.00	0.00	0.00	0.06	0.00	0.00	0.00	0.00	0.00	0.00	0.00	0.00	0.00	0.00	0.00	
105	0.00	0.00	0.00	0.00	0.05	0.00	0.01	0.14	0.00	0.01	0.00	0.04	0.00	0.00	0.00	0.00	0.00	0.00	0.00	0.00	0.00	0.00	0.00	0.00	
106	0.00	0.07	0.00	0.04	0.00	0.01	0.02	0.04	0.06	0.01	0.00	0.00	0.00	0.00	0.00	0.00	0.00	0.01	0.00	0.00	0.00	0.00	0.00	0.00	
107	0.00	0.23	0.00	0.00	0.00	0.00	0.01	0.04	0.03	0.00	0.00	0.00	0.00	0.00	0.00	0.00	0.00	0.00	0.00	0.11	0.00	0.00	0.00	0.00	
108	0.00	0.03	0.00	0.00	0.03	0.00	0.02	0.03	0.00	0.00	0.00	0.00	0.00	0.00	0.00	0.00	0.00	0.00	0.00	0.00	0.00	0.02	0.09	0.00	
109	0.00	0.01	0.00	0.00	0.00	0.00	0.00	0.04	0.00	0.00	0.00	0.00	0.00	0.00	0.00	0.00	0.00	0.00	0.06	0.00	0.00	0.00	0.02	0.00	
110	0.00	0.02	0.00	0.00	0.00	0.00	0.01	0.04	0.00	0.00	0.00	0.00	0.00	0.05	0.00	0.00	0.00	0.03	0.00	0.00	0.00	0.00	0.00	0.00	M-PER/MSD 110-115
111	0.00	0.01	0.00	0.00	0.01	0.03	0.02	0.04	0.00	0.00	0.00	0.00	0.00	0.00	0.00	0.00	0.00	0.00	0.00	0.00	0.00	0.00	0.00	0.00	
112	0.00	0.00	0.00	0.00	0.00	0.00	0.00	0.04	0.00	0.00	0.00	0.00	0.00	0.00	0.00	0.00	0.00	0.00	0.00	0.00	0.00	0.00	0.00	0.00	
113	0.00	0.00	0.00	0.00	0.00	0.00	0.00	0.02	0.00	0.00	0.00	0.07	0.00	0.00	0.00	0.00	0.00	0.00	0.00	0.00	0.00	0.00	0.00	0.00	
114	0.00	0.00	0.00	0.01	0.01	0.01	0.02	0.02	0.00	0.00	0.00	0.00	0.00	0.00	0.19	0.00	0.00	0.00	0.00	0.15	0.00	0.00	0.00	0.00	
115	0.00	0.00	0.03	0.01	0.00	0.00	0.00	0.02	0.00	0.00	0.03	0.00	0.00	0.00	0.00	0.00	0.00	0.00	0.00	0.00	0.00	0.00	0.00	0.00	

Color scale: Mean OD_{450} (0, 0.5, 1.0, 1.5, 2.0, 2.5, 3.0, 3.5, 4.0)

Env SIV_{mac251} overlapping peptides (1-116)

Figure S4

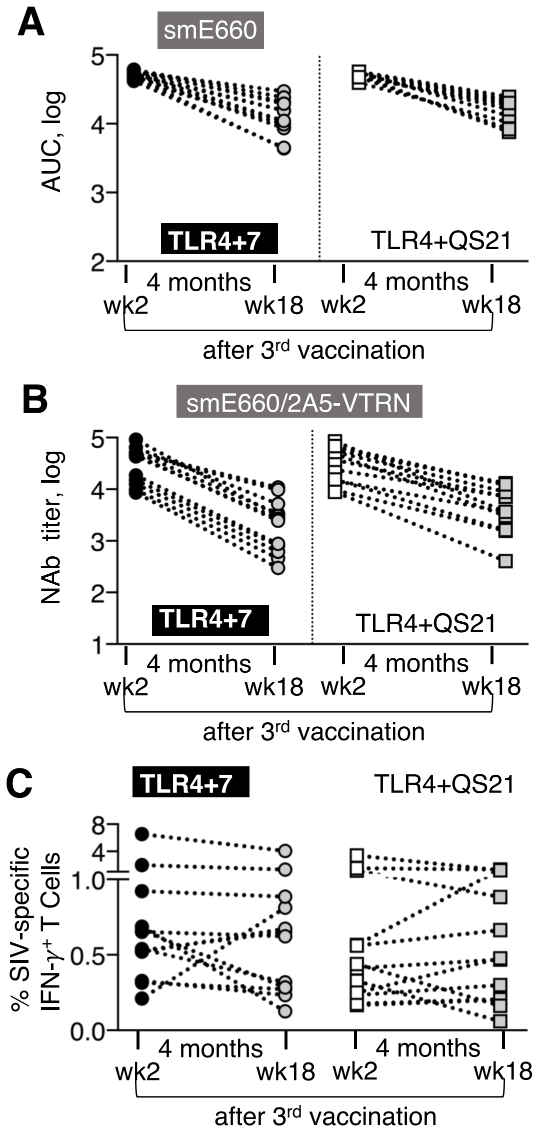
A
smE660
AUC, log
5
4
3
2
TLR4+7
TLR4+QS21
4 months
4 months
wk2
wk18
wk2
wk18
after 3rd vaccination
B
smE660/2A5-VTRN
NAb titer, log
5
4
3
2
1
TLR4+7
TLR4+QS21
4 months
4 months
wk2
wk18
wk2
wk18
after 3rd vaccination
C
TLR4+7
TLR4+QS21
% SIV-specific
IFN-γ+ T Cells
8
4
1.0
0.5
0.0
4 months
4 months
wk2
wk18
wk2
wk18
after 3rd vaccination

Figure S5

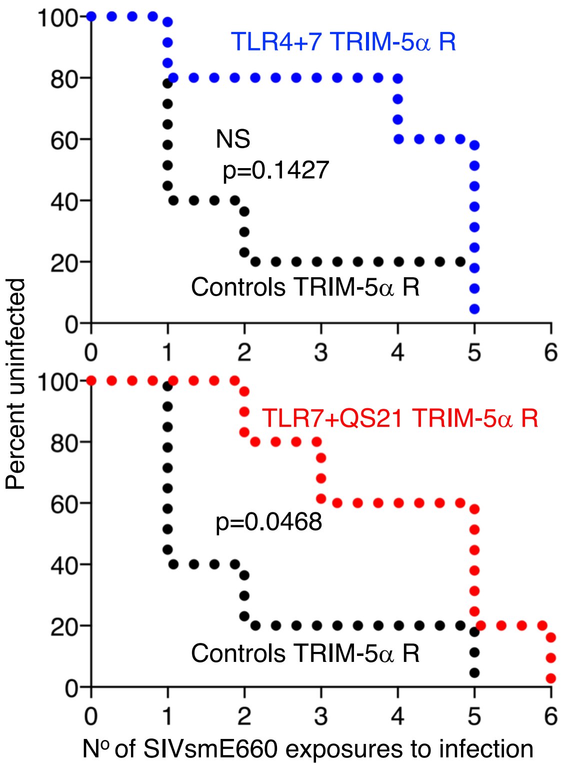
TLR4+7 TRIM-5α R
NS
p=0.1427
Controls TRIM-5α R
TLR7+QS21 TRIM-5α R
p=0.0468
Controls TRIM-5α R
Percent uninfected
Nº of SIVsmE660 exposures to infection
100
80
60
40
20
0
0
1
2
3
4
5
6

Figure S6

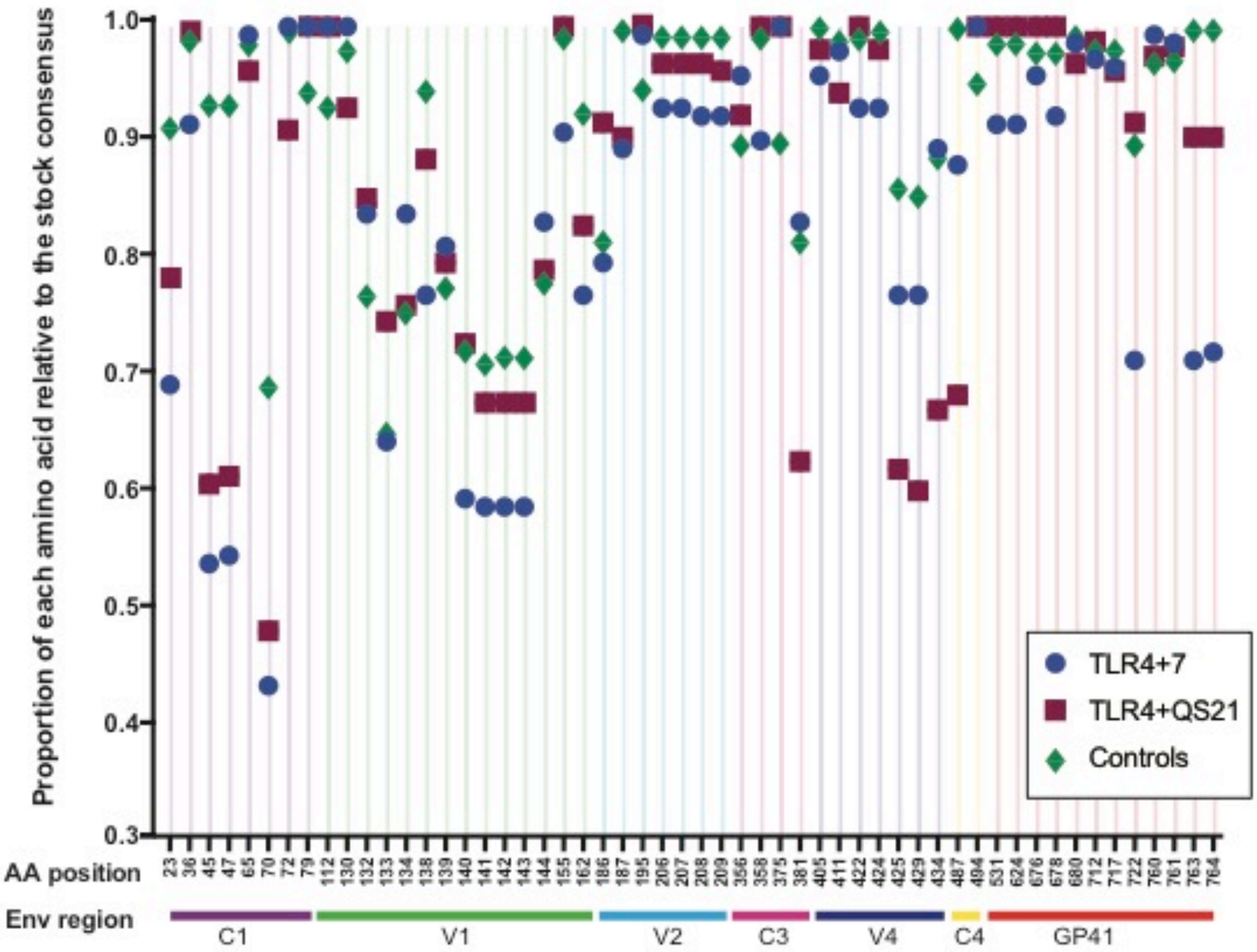

Proportion of each amino acid relative to the stock consensus
1.0
0.9
0.8
0.7
0.6
0.5
0.4
0.3
AA position
23 36 45 47 65 70 72 79 112 130 132 133 134 138 139 140 141 142 143 144 155 162 186 187 195 206 207 208 209 356 358 375 381 405 411 422 424 425 429 434 487 494 531 624 676 678 680 712 717 722 760 761 763 764
Env region
C1
V1
V2
C3
V4
C4
GP41
TLR4+7
TLR4+QS21
Controls

Figure S7

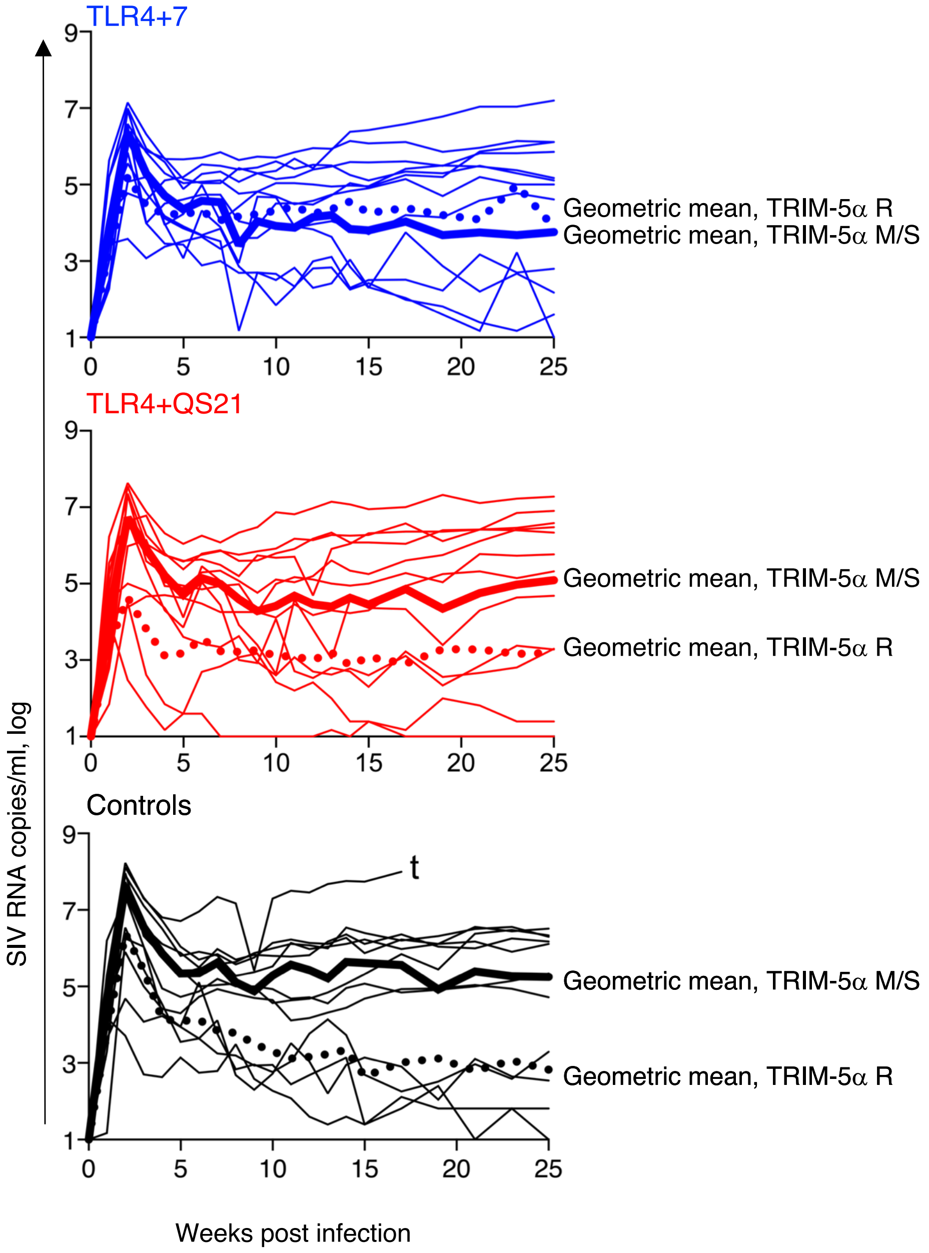
TLR4+7
Geometric mean, TRIM-5α R
Geometric mean, TRIM-5α M/S
TLR4+QS21
Geometric mean, TRIM-5α M/S
Geometric mean, TRIM-5α R
Controls
t
Geometric mean, TRIM-5α M/S
Geometric mean, TRIM-5α R
SIV RNA copies/ml, log
Weeks post infection
1
3
5
7
9
0
5
10
15
20
25

Figure S8

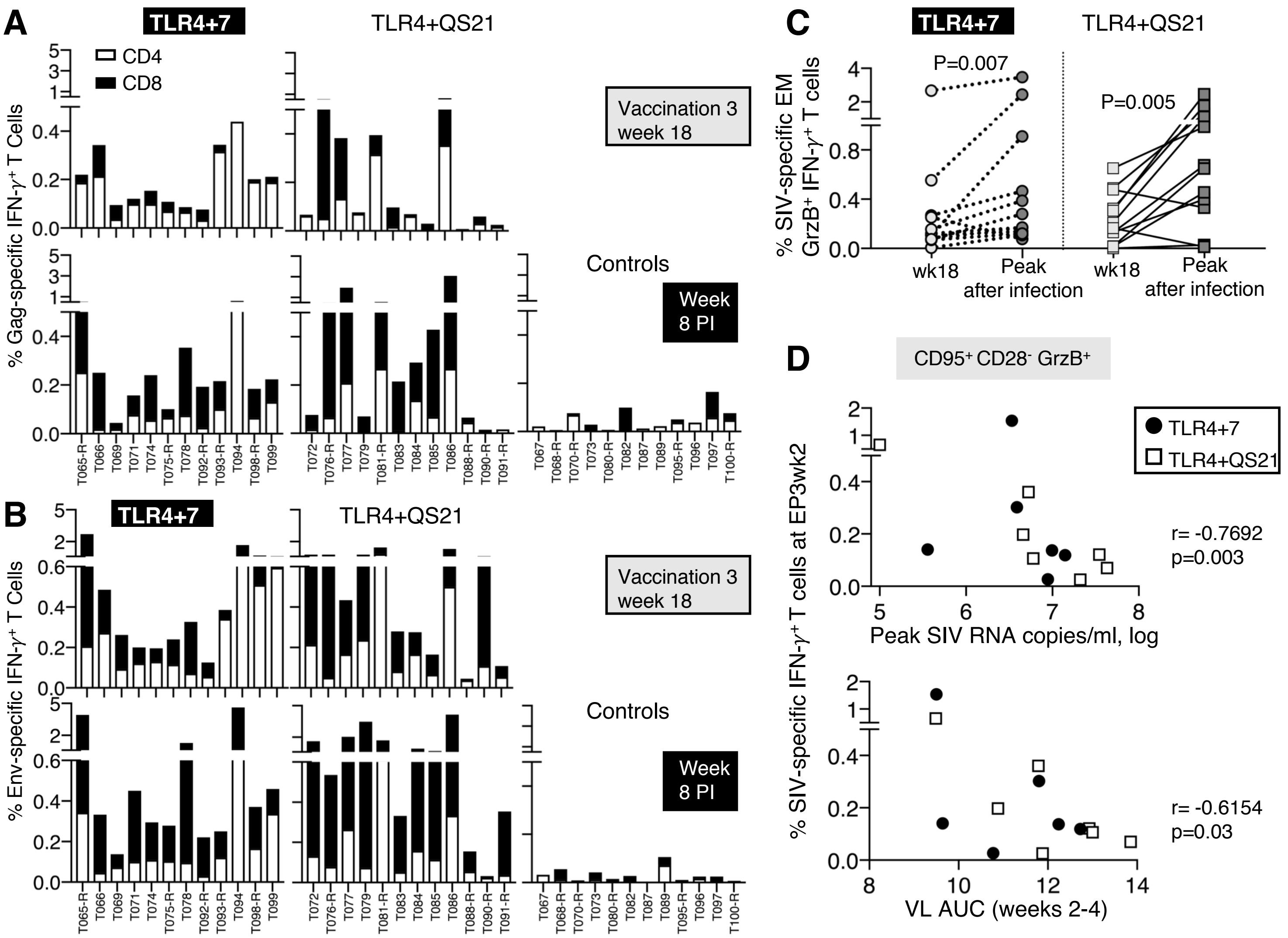
A
TLR4+7
TLR4+QS21
CD4
CD8
Vaccination 3
week 18
Controls
Week
8 PI
% Gag-specific IFN-γ+ T Cells
T065-R T066 T069 T071 T074 T075-R T078 T092-R T093-R T094 T098-R T099
T072 T076-R T077 T079 T081-R T083 T084 T085 T086 T088-R T090-R T091-R
T067 T068-R T070-R T073 T080-R T082 T087 T089 T095-R T096 T097 T100-R
B
TLR4+7
TLR4+QS21
Vaccination 3
week 18
Controls
Week
8 PI
% Env-specific IFN-γ+ T Cells
C
TLR4+7
TLR4+QS21
P=0.007
P=0.005
% SIV-specific EM GrzB+ IFN-γ+ T cells
wk18
Peak
after infection
D
CD95+ CD28- GrzB+
TLR4+7
TLR4+QS21
% SIV-specific IFN-γ+ T cells at EP3wk2
Peak SIV RNA copies/ml, log
r= -0.7692
p=0.003
VL AUC (weeks 2-4)
r= -0.6154
p=0.03
